# *HLA-DRB1* allelic epitopes that associate with autoimmune disease risk or protection activate reciprocal macrophage polarization

**DOI:** 10.1038/s41598-021-82195-3

**Published:** 2021-01-28

**Authors:** Vincent van Drongelen, Bruna Miglioranza Scavuzzi, Sarah Veloso Nogueira, Frederick W. Miller, Amr H. Sawalha, Joseph Holoshitz

**Affiliations:** 1 Department of Internal Medicine, University of Michigan, Ann Arbor, MI 48109 USA; 2 Environmental Autoimmunity Group, National Institute of Environmental Health Sciences, Research Triangle Park, Durham, NC 27709 USA; 3 Present Address: Departments of Pediatrics and Internal Medicine, University of Pittsburgh, Pittsburgh, PA 15224 USA

**Keywords:** Autoimmunity, Immunogenetics

## Abstract

Associations between particular human leukocyte antigen (HLA) alleles and susceptibility to—or protection from—autoimmune diseases have been long observed. Allele-specific antigen presentation (AP) has been widely proposed as a culprit, but it is unclear whether HLA molecules might also have non-AP, disease-modulating effects. Here we demonstrate differential macrophage activation by *HLA-DRB1* alleles known to associate with autoimmune disease risk or protection with resultant polarization of pro-inflammatory (“M1”) versus anti-inflammatory (“M2”) macrophages, respectively. RNA-sequencing analyses of in vitro-polarized macrophages in the presence of AP-incompetent short synthetic peptides corresponding to the third allelic hypervariable regions coded by those two *HLA-DRB1* alleles showed reciprocal activation of pro- versus anti-inflammatory transcriptomes, with implication of corresponding gene ontologies and upstream regulators. These results identify a previously unrecognized mechanism of differential immune modulation by short *HLA-DRB1*-coded allelic epitopes independent of AP, and could shed new light on the mechanistic basis of HLA-disease association.

## Introduction

It has been long observed that certain human leukocyte antigen (HLA) alleles confer higher risk for autoimmune diseases, while other alleles provide protective effects^1^. The molecular mechanisms underlying these associations are incompletely understood. Modeled on the major histocompatibility complex (MHC)-restricted antigen presentation (AP) paradigm^2,3^, it has been hypothesized that HLA-associated diseases are triggered by presentation of self-antigens—or foreign antigens that cross-react with self by HLA-coded molecules^4–7^. It is worth noting, however, that the identity of the target antigens remains unknown in many HLA-associated conditions.

Compelling evidence of antigen-specific immune response exists in several HLA-associated conditions, such as celiac disease (Reviewed in^8^), or type I diabetes mellitus (Reviewed in^9^). In many other diseases, however, a candidate target antigen has not been yet identified, or AP might not be the sole mechanistic basis for their HLA associations, as previously discussed^10,11^. Two emblematic HLA-associated diseases, ankylosing spondylitis (AS) and rheumatoid arthritis (RA) illustrate the quandary.

AS associates strongly with particular HLA-B27 subtypes^12,13^. While the search for arthritogenic peptides that these class I HLA antigens putatively present is underway, a recent study found no qualitative differences in peptide binding preferences among HLA-B27 subtypes that associate with AS, versus disease-unassociated subtypes^14^. Additionally, in a rat model of AS, class II-restricted CD4^+^ T cells, rather than class I-restricted CD8^+^ T cells appear to be involved in disease pathogenesis (Reviewed in^15^). Moreover, it has been recently established that independent of their putative disease-enhancing AP properties, AS-associated HLA-B27 molecules have a unique propensity to activate endoplasmic reticulum stress and misfold intracellularly, thereby triggering overproduction of pro-arthritogenic cytokines^15^.

In RA, the majority of affected individuals carry *HLA-DRB1* alleles that code for a susceptibility (or ‘shared’) epitope (SE) motif QKRAA, QRRAA, or RRRAA in position 70–74 of the DRβ chain^16^. However, the association is not disease-specific. SE-coding *HLA-DRB1* alleles have also been found to associate with type 1 diabetes, autoimmune hepatitis, polymyalgia rheumatica, temporal arteritis and Parkinson’s disease^17–21^, among other conditions. Further, the SE has been found to be a risk factor for erosive bone damage in nosologically-distinct conditions, such as periodontal disease, systemic lupus erythematosus and psoriatic arthritis^22–26^. Additionally, while AP by SE-expressing HLA-DR molecules has been long postulated to be a disease-susceptibility mechanism in RA, only minor overlaps were found among the repertoires of peptides eluted from different SE-expressing HLA-DR molecules (encoded by either DRB1*01:01; DRB1*04:01, or DRB1*10:01). Notably, comparable overlaps were found with a non-SE-expressing (DRB1*15:01) molecule^27^.

An inverse disease association exists with *HLA-DRB1* alleles that encode a protective epitope (PE) motif 70-DERAA-74 (at the exact same region of the DRβ chain as the SE). Those alleles significantly reduce the risk for RA^28–31^, and many other autoimmune conditions, including systemic lupus erythematosus, mixed connective tissue disease, progressive systemic sclerosis, antineutrophil cytoplasmic antibodies (ANCA)-positive vasculitis, narcolepsy and myasthenia gravis^32–37^.

Thus, both the SE and PE modulate disease risk in a wide range of conditions that do not share a common putative target antigen, pathogenesis, or target tissues. To determine whether the SE and PE, aside from their AP function, may also possess AP-independent effects, here we compared their respective impacts on macrophage activation, given the pivotal role that these cells play in autoimmunity^38–42^. The findings demonstrate that primary bone marrow macrophages from naïve transgenic mice that constitutively express on their cell surface SE-positive HLA-DR molecules are polarized preferentially toward pro-inflammatory (“M1”) macrophage, while macrophages from transgenic mice that express PE-positive HLA-DR molecules show enhanced anti-inflammatory (“M2”) polarization.

To exclude the possibility of AP of self- or tissue culture-derived antigens by the transgenic HLA-DR molecules, and to map the functional epitopes, we studied AP-incompetent soluble synthetic 15-mer peptides corresponding to the third allelic hypervariable regions (TAHRs) coded by these two *HLA-DRB1* alleles. Using an RNA sequencing (RNA-seq) approach, we demonstrate here that consistent with the above findings, exposure of macrophages to SE- or PE-expressing peptides activates distinct transcriptomes. A SE-expressing peptide activates genes, biological processes and upstream regulators known to mediate pro-M1, pro-inflammation or pro-autoimmune disease effects, whereas a PE-expressing peptide facilitates activation of genes, biological processes and upstream regulators that are known to mediate pro-M2, anti-inflammatory, or anti-autoimmune disease effects. Thus, gene products coded by *HLA-DRB1* alleles that are known to associate with autoimmune disease susceptibility versus protection activate, respectively, pro-inflammatory or anti-inflammatory pathways in an AP-independent fashion.

## Results

### HLA-DRB1 allele-specific differential macrophage polarization in transgenic mice

**Figure 1:**
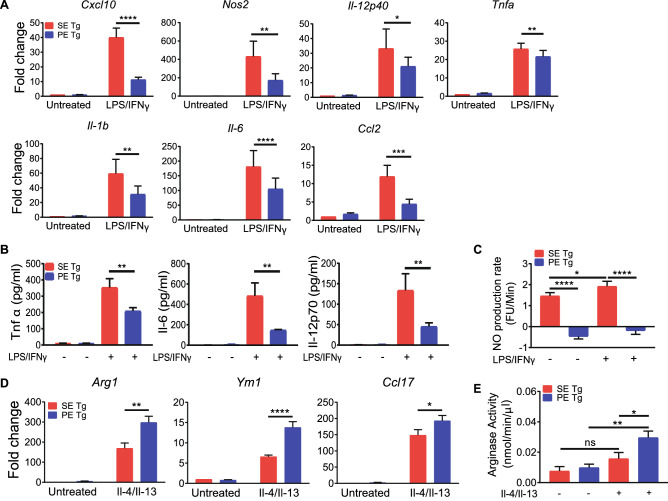
Differential in vitro macrophage polarization in SE Tg and PE Tg BMDMs. For M1 polarization, BMDMs were treated with LPS (1 ng/ml) + IFNγ (20 ng/ml) for 24 h. M2 polarization was induced with Il-4 (10 ng/ml) + Il-13 (10 ng/ml) for 24 h. (**A**) qPCR for M1-associated genes. Data represent mean + SEM of 3–5 independent experiments. (**B**) ELISA for M1-associated cytokines. Data represent mean + SEM of 3 independent experiments. (**C**) NO production by BMDMs under M1 polarization conditions. Data represent mean + SEM of 8 replicates from 2 independent experiments. (**D**) qPCR for M2-associated genes. Data represent mean + SEM of 3–4 independent experiments. (**E**) Arginase activity in BMDMs under M2 polarization conditions. Data represent mean + SEM of 3 independent experiments. 2-way ANOVA, *P < 0.05, **P < 0.01, ***P < 0.001, ****P < 0.0001.

To determine whether autoimmune disease risk or protective *HLA-DRB1* alleles have distinct effects on macrophage polarization we first studied ex vivo primary bone marrow-derived macrophages (BMDMs) isolated from transgenic mice^43,44^ that express human HLA-DRβ chains coded by the DRB1*04:02 allele with a PE (70-DERAA-74) sequence in the TAHR (“PE Tg”), versus BMDMs from mice of the same genetic background expressing a SE (70-QKRAA-74) sequence, encoded by the RA-susceptibility allele DRB1*04:01 (“SE Tg”). The TAHRs of the two transgenic mouse lines differ by only 3 amino acid residues. As shown in Fig. 1, under M1-polarizing culture conditions, SE Tg-derived BMDMs expressed higher levels of the M1 gene markers *Cxcl10*, *Nos2*, *Il*-*12p40*, *Il-1b, Tnfa*, *Il-6* and *Ccl2* compared to PE Tg-derived BMDMs (Fig. 1A). Additionally, SE Tg-derived BMDMs produced Il-6, Tnfα, and Il-12p70 proteins at significantly higher levels than PE Tg-derived BMDMs (Fig. 1B). SE Tg BMDMs also showed higher baseline levels of nitric oxide (NO) production with a significant increase under M1 polarizing culture conditions, whereas PE Tg-derived BMDMs did not produce any NO in either culture conditions (Fig. 1C). A mirror-image pattern was found under M2-polarizing culture conditions: PE Tg BMDMs demonstrated higher expression of the M2 gene markers *Arg1*, *Ym1* and *Ccl17* relative to SE Tg BMDMs (Fig. 1D). Consistent with the above findings, and with the role of arginase in M2 polarization^45^, a significantly increased arginase activity under M2 polarizing conditions was found in BMDMs from PE Tg, compared to SE Tg (Fig. 1E).

**Figure 2:**
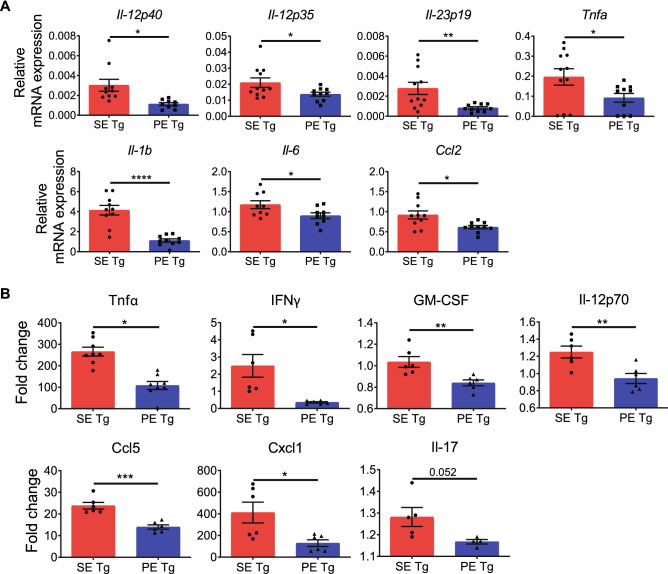
Differential in vivo macrophage polarization in SE Tg and PE Tg mice. To induce M1 polarization in vivo, mice were injected i.p. with LPS (500 µg/kg). Peritoneal macrophages and serum were collected after 4 h. (**A**) qPCR for mRNA expression of M1 marker genes in peritoneal macrophages. n = 8–11. (**B**) ELISA for serum cytokine levels. n = 5–8. Results are compiled data from 3 experiments. Mean ± SEM, unpaired *t* test with welch correction, *P < 0.05, **P < 0.01, ***P < 0.001, ****P < 0.0001.

*HLA-DRB1* allele-based differential macrophage polarization was found in vivo as well. As shown in Fig. 2A, intra peritoneal (i.p.) administration of an M1-polarizing agent (LPS) significantly increased gene expression of M1 markers *Il12p40*, *Il12p35*, *Il23p19*, *Il-1b*, *Tnfa*, *Ccl2* and *Il-6* in peritoneal macrophages from SE Tg, compared to PE Tg mice. Additionally, SE Tg mice produced higher serum levels of various pro-inflammatory, M1-associated cytokines, including Il-12, TNFα, GM-CSF and IFNγ (Fig. 2B).

### Signal transduction pathways

**Figure 3:**
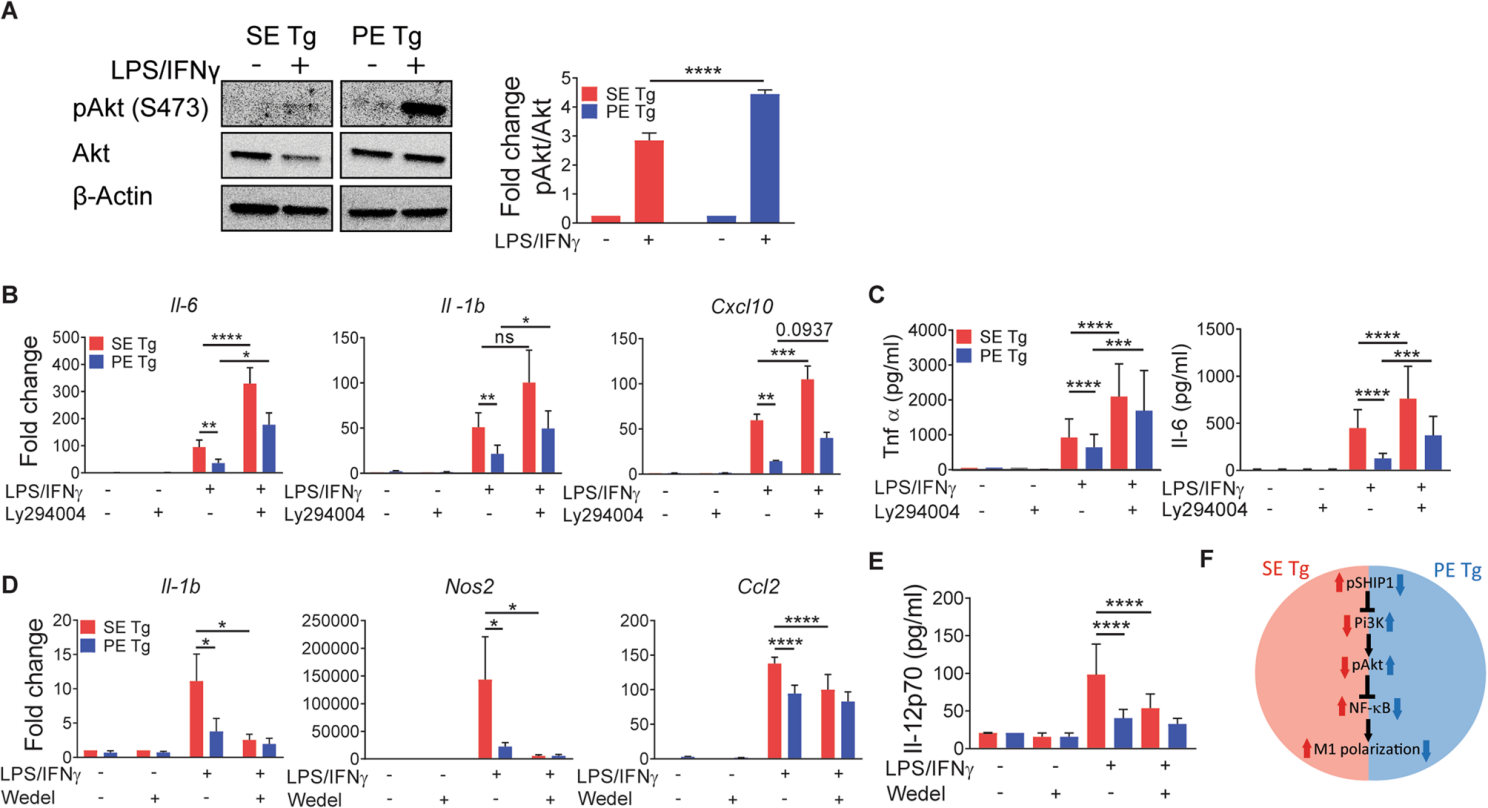
Involvement of signaling pathways in SE Tg and PE Tg BMDMs under M1 polarization conditions. M1 polarization in BMDMs was performed using the conditions described in Fig. 1. (**A**) Immunoblot for pAKt (Ser473) and Akt 15 min. after exposure of cells to M1 polarization conditions. Quantification data represent mean + SEM of 3 independent experiments. (**B**–**E**) BMDMs were pre-treated with Ly294002 (5 µM) (**B**,**C**), or wedelolactone (10 µM) (**D**,**E**) for 1 h., followed by incubation in M1 polarization conditions as in (**A**). (**B**,**D**) qPCR-based determination of M1 gene marker expression levels. (**C** and **E**) ELISA for Tnfα or Il-6 (**C**), or IL12p70 (**E**). (**F**) A proposed model of signaling pathway involvement under M1 polarizing conditions. Data represent mean and SEM of 3–5 independent experiments. Statistics: within group comparisons, paired *t* test; between groups comparison, 2-way ANOVA, *P < 0.05, **P < 0.01, ***P < 0.001, ****P < 0.0001.

To better understand the mechanisms involved in *HLA-DRB1* allele-based macrophage polarization predilections, we sought to determine whether signaling events are involved. To this end we focused on the Akt axis, known to play a pivotal role in M1 versus M2 macrophage polarization^46,47^. As Fig. 3 shows, under M1 polarizing conditions, BMDMs from PE Tg mice displayed significantly increased Akt phosphorylation compared to BMDMs from SE Tg mice (Fig. 3A). Ly294002-mediated inhibition of Pi3K, an upstream regulator of Akt, allowed significant rescue of the expression of M1 gene markers *Il-1b* and *Il-6* (Fig. 3B), as well as Il-6 and Tnfα cytokine levels (Fig. 3C). To better characterize the upstream signaling events that impact Akt activation, we measured phosphorylation of two Pi3K regulating phosphatases: PTEN and SHIP1 under M1 polarizing conditions. PTEN phosphorylation levels were not different between SE Tg and PE Tg BMDMs (Supplemental Fig. S1A). However, SHIP1, showed significantly lower phosphorylation levels in PE Tg BMDMs compared to SE Tg (Supplemental Fig. S1B).

Another important signaling mechanism in M1 polarization is NF-κB^48^, mapped downstream of Akt^49^. We therefore asked whether NF-κB plays a role in *HLA-DRB1*-associated macrophage polarization. Under M1 polarizing conditions, inhibition of NF-κB decreased expression of M1 gene markers *Il-1b*, *Nos2* and *Ccl2*, as well as the M1 cytokine Il-12p70 in SE Tg BMDMs, but not in PE Tg BMDMs (Fig. 3D,E). Thus, taken together, we propose that the diminished M1 polarizability of PE Tg BMDMs is secondary to increased Akt activation, previously shown to inhibit NF-κB signaling^46^. We further propose that increased SHIP1 activity in SE Tg BMDMs results in reduced Akt activation, leading to enhanced NF-κB activity, which, in turn leads to increased M1 polarization, consistent with previous studies^46^. A proposed model of the signaling pathways involved in *HLA-DRB1* allele-specific M1 macrophage polarization is shown in Fig. 3F.

**Figure 4:**
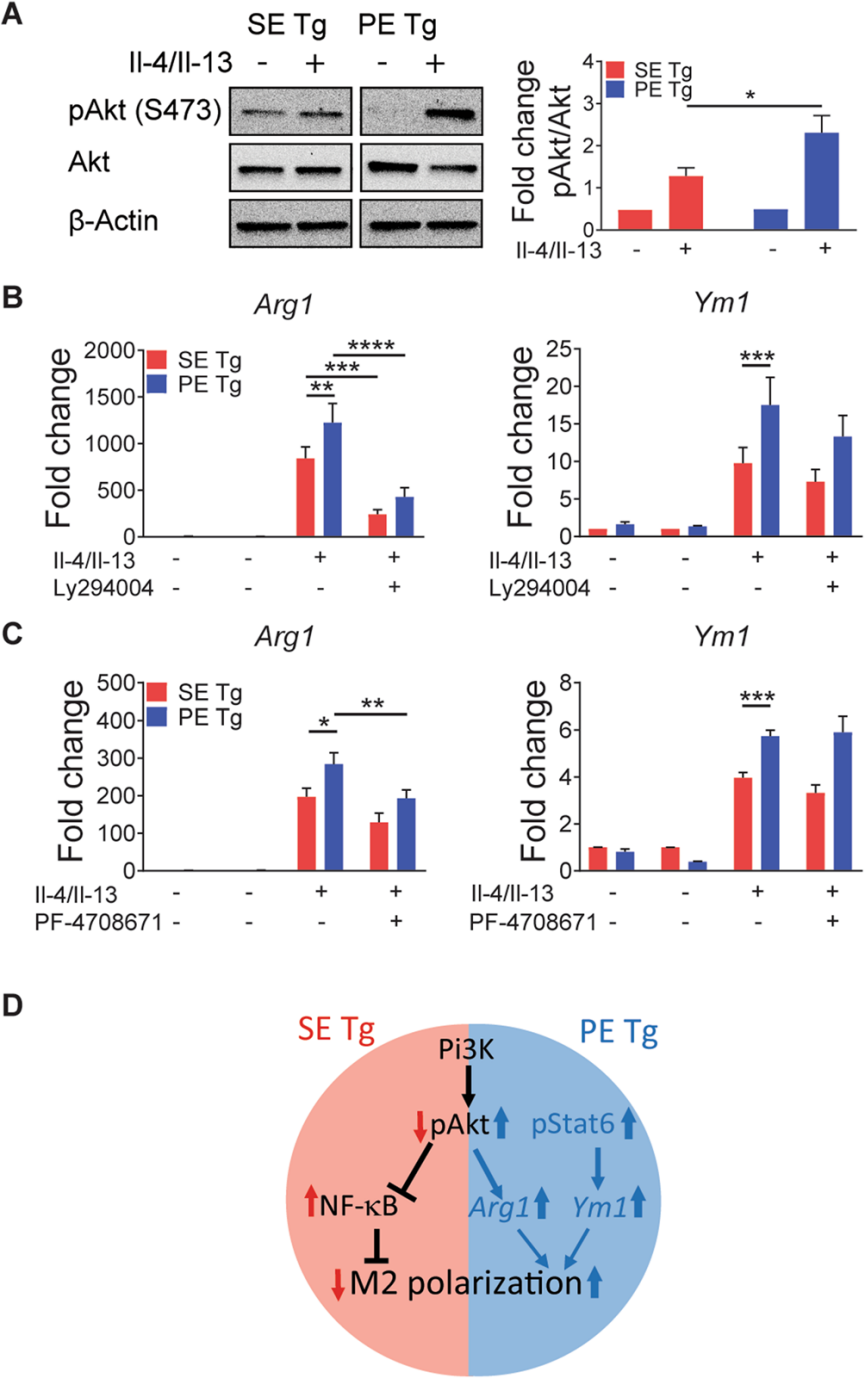
Involvement of signaling pathways in SE Tg and PE Tg BMDMs under M2 polarization conditions. M2 polarization of BMDMs was performed as in Fig. 1. (**A**) Immunoblotting for pAKt (Ser473) and Akt in BMDMs 10 min after exposure of cells to M2 polarization conditions. Quantification data represent mean + SEM of 3 independent experiments. (**B**,**C**) qPCR analysis for M2 gene markers *Arg1* and *Ym1* expression in BMDMs pre-treated with Ly294002 (5 µM) (**B**), or PF4708671 (10 µM) (**C**) for 1 h, followed by M2 polarization for 20 h. Data represent mean + SEM of 3 independent experiments. Statistics: within group comparisons, paired *t* test; between groups comparison, 2-way ANOVA, *P < 0.05, **P < 0.01, ***P < 0.001, ****P < 0.0001. (**D**) A proposed model of signaling pathway involvement under M2 polarizing conditions.

Under M2 polarization conditions, significantly higher Akt phosphorylation was found in PE Tg BMDMs compared to SE Tg BMDMs (Fig. 4A). Modulation of the Akt signaling pathway through inhibition of Pi3K (upstream of Akt), or p70S6K (downstream of Akt), significantly suppressed expression of the M2 gene marker *Arg1* (Fig. 4B,C, respectively). Noteworthy, another M2 gene marker *Ym1* was not affected by either Pi3K or p70S6K inhibitors, suggesting that, different from *Arg1*, increased *Ym1* gene expression in PE Tg BMDMs is Akt-independent. The possibility that *Ym1* gene expression in PE Tg BMDMs is controlled by Stat6, as previously reported in other cells^50^, is consistent with significantly higher Stat6 phosphorylation levels under M2 polarization conditions in PE Tg, compared to SE Tg BMDMs (Supplemental Fig. S2). A proposed model of the signaling pathways involved in *HLA-DRB1* allele-specific M2 macrophage polarization is shown in Fig. 4D.

### HLA-DRB1 allele-specific transcriptome activation

To more conclusively determine whether the differential effects of the two *HLA-DRB1* alleles may be AP-independent and to determine whether their effects could be mapped to the TAHR, we used AP-incompetent 15-mer synthetic peptides corresponding to the TAHR of the DRβ chain.

To explore the feasibility of this approach, we first quantified expression levels of macrophage polarization marker genes in RAW 264.7 mouse macrophages following exposure to 15-mer peptides designated “65–79*SE” or “65–79*PE”, corresponding to amino acid residues 65–79 (TAHR) coded, respectively, by susceptibility (HLA-DRB1*04:01) or protective (e.g. HLA-DRB1*04:02, DRB1*13:01, or DRB1*13:02) alleles. The 65–79*SE and 65–79*PE peptides differ by 3 amino acid residues, including only 2 substitutions in the 70–74 region (QKRAA versus DERAA).

The rationale for studying these 15-mer peptides is based on previous studies, which demonstrated that 65–79*SE and its core 5-mer motif QKRAA activate pro-arthritogenic signal transduction and cell activation events^51–55^. These TAHR peptides were also found to carry out important arthritis-modulating functions. For example, soluble 65–79*SE and peptidomimetics of the QKRAA motif have been found to be arthritogenic in mice^51,56^, whereas 65–79*PE has been shown to dominantly protect against arthritis in mice carrying a permissive haplotype^57^, consistent with the findings that allele HLA-DRB1*04:02 is dominantly protective against arthritis in both mice^44^ and humans^58^. Additionally, Q70D substitution has a significant arthritis-protective effect in mice^59^, consistent with the role of D70 as a key PE residue for RA protection^60^.

As shown in Supplemental Figure S3, quantitative RT-PCR analysis confirmed that under M1-polarizing conditions, 65–79*SE, but not the 65–79*PE, activated transcription of the M1-marker genes *Nos1*, *Cxcl10*, *Ccl2* and *Il-1b* (Supplemental Fig. S3A). Under M2 polarizing conditions, 65–79*PE selectively upregulated expression of the M2 marker gene *Mgl2* (Supplemental Fig. S3B). Thus, consistent with the findings in transgenic mouse BMDMs, 15-mer peptides 65–79*SE and 65–79*PE, corresponding to the TAHRs coded by *DRB1* alleles that confer autoimmune disease risk or protection, respectively, recapitulated the differentially induced expression of M1 versus M2 macrophage polarization gene markers in an epitope-specific fashion.

**Figure 5:**
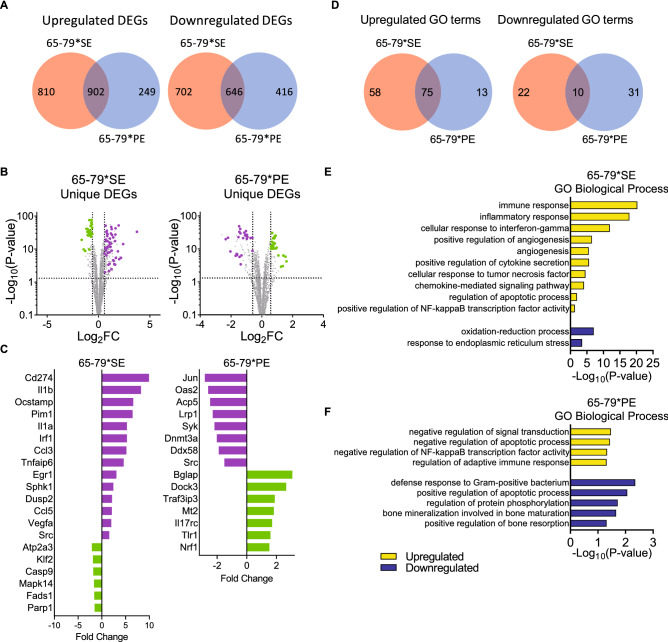
Transcription modulation by 65–79*SE and 65–79*PE under M1 polarizing conditions. Mouse RAW 264.7 macrophages were incubated for 3 days with IFNγ (5 ng/ml) in the presence or absence of 100 μg/ml 65–79*SE or 65–79*PE and RNA-seq analysis was performed on isolated RNA. Data are from 6 biological replicates in 2 independent experiments. (**A**) Venn diagrams showing upregulated and downregulated DEGs (P adjusted < 0.05, fold change > 1.5). (**B**) Volcano plots for unique DEGs for 65–79*SE or 65–79*PE. Purple dots denote pro-RA-associated genes; green dots denote RA-protective genes. (**C**) Selected notable genes unique for 65–79*SE or 65–79*PE with known RA-related functions. Purple bars denote pro-RA-associated genes; green bars denote RA-protective genes. (**D**) Venn diagrams of overlapping and unique GO terms derived from upregulated and downregulated DEGs in (**A**). (**E, F**) Selected GO Biologic Processes for up- and downregulated DEGs by 65–79*SE (**E**) or 65–79*PE (**F**).

We next used an RNA-seq approach to characterize the broader transcriptional effects of the two TAHRs in mouse RAW 264.7 macrophages stimulated with 65–79*SE or 65–79*PE. Under M1 polarizing conditions (Fig. 5) the two TAHR peptides had a distinct effect on the number of upregulated and downregulated differentially expressed genes (DEGs) (Fig. 5A, Data File S1A). Among the unique upregulated genes induced by 65–79*SE in M1-polarizing conditions (Fig. 5B,C, Supplemental Table S1, Data File S2A) were many RA disease marker—or risk factor—genes (e.g. *Stat3*, *Cd44*, *Traf1*, *Tnfaip3*), genes that encode confirmed or proposed therapeutic targets (e.g. *Jak1*, *Jak3*, *Stat3*, *Ccr1*, *Cxcr4*, *Mmp14*), and genes involved in autoimmune disease pathogenic mechanisms, such as NF-κB activation (e.g. *Relb*, *Kpna4*, *Vav1*), angiogenesis (*Dusp4*, *Pgf*, *Egr1*, *Vegfc*, *Vegfa*, *Hbegf*, *Hmga1*), Th17 polarization (e.g. *Dusp4*, *Runx1*, *Hbegf*), osteoclastogenesis (e.g. *Atf4*, *Adam8*, *Dcstamp*, *Cxcr1*), or M1 polarization (*Klf6*). Conversely, 65–79*SE downregulated many anti-inflammatory (e.g. *Akap1*, *Casp9*, *Cyp51*, *Hsd17b4*, *Scarb2*), anti-angiogenesis (e.g. *Ctdsp1*, *Lyl1*, *Mapk14*, *Patz1*, *Ywhab*), NF-κB inhibitor (e.g. *Atp2a3*, *Bag2*), and pro-M2 (e.g. *Fads1*, *Klf2*, *Lpcat3*, *Pon2*, *Ywhab*) genes. Representative modulated genes are shown in Fig. 5C. A list of notable genes, along with annotations and statistical significances, is shown in Supplemental Table S1. A complete list of unique 65–79*SE-modulated DEGs is shown in Data File S2A.

A diametrically opposite gene transcription pattern under M1-polarizing conditions was found with 65–79*PE (Fig. 5B, Data File S2B). It showed uniquely upregulated anti-inflammatory (e.g. *Mt2*, *Il17rc*, *Havcr2*, *Ogg1*, *Nrf1*, *Stk10*, *Pdcd2*), anti-angiogenesis (e.g. *Flip1l*, *Fyn*, *Htatip2*), anti-bone resorption (e.g. *Def6*, *Gpr65*, *Bglap*, *Fbxl12*), anti-oxidative (e.g. *Mt2*, *Pycr1*, *Stc2*, *Dock3*), and pro-M2 or anti-M1 (e.g. *Tlr1*, *Nrf1*, *Themis2*) genes. Conversely, unique downregulated genes by 65–79*PE included many pro-osteoclastogenic (e.g. *Atp6v0d2*, *Dnmt3a*, *Syk*, *Ckb*, *Tspan5*, *Acp5*, *Src*), pro-angiogenesis (e.g. *St3gal1*, *Glul*, *Epn2*, *F7*, *Arhgap24*), pro-arthritogenic (e.g. *Syk*, *Jun*, *Tnfrsf9*, *F10*, *Arhgap24*, *Adamts7*), and NF-κB pathway-activating (e.g. *Sh3kbp1*, *Ddx58*, *F7*) genes. Representative genes are shown in Fig. 5C. A list of notable genes, along with annotations and statistical significances, is shown in Supplemental Table S1. A complete list of unique 65–79*PE-modulated DEGs is shown in Data File S2B.

Gene ontology (GO) analysis (Fig. 5D–F) revealed many GO processes that involve cytokine/chemokine signaling, innate immune response and positive regulation of NF-κB activity in macrophages stimulated with 65–79*SE under M1 polarizing conditions (Fig. 5E, Data File S1C). By contrast, GO terms for genes upregulated by 65–79*PE in M1 polarizing conditions included processes involving inhibitory effects on signal transduction, protein phosphorylation, adaptive immune response and NF-κB (Fig. 5F, Data File S1D). Conversely, GO terms for genes downregulated by 65–79*PE included bone mineralization and resorption, known as important effector mechanisms in RA pathogenesis. In summary, under M1 polarizing conditions, the PE-expressing TAHR activated an anti-inflammatory or anti-RA transcriptome, whereas the SE-expressing TAHR activated a pro-inflammatory, pro-RA transcriptome.

To determine whether TAHR polarizing effects could be found in human cells as well, we performed RNA-seq analysis in human THP-1 macrophages under M1 polarizing conditions, and found remarkable similarities between the two species (Supplemental Fig. S4, Data File S3). For example, 100 (22%) of the 446 DEGs upregulated by 65–79*SE in human THP-1 cells were also upregulated by this TAHR in mouse RAW 264.7 macrophages (Supplemental Fig. S4A, Data File S3A,B). Moreover, the top-ranked 60 DEGs that were found to be upregulated by 65–79*SE in both species were searched in PubMed, and 68% of them were found to be positively associated with RA disease risk or pathogenesis (Supplemental Fig. S4B, Supplemental Table S1, Data File S3B). GO analysis revealed many shared biologic processes (Supplemental Fig. S4C, Data File S3C). Moreover, Kyoto Encyclopedia of Genes and Genomes (KEGG) pathways analysis (Supplemental Fig. S4D, Data File S3C) revealed a high level of correspondence between mouse and human macrophages in many relevant pathways, and identified the KEGG term Rheumatoid Arthritis as the top-ranked pathway in the THP-1 list.

**Figure 6:**
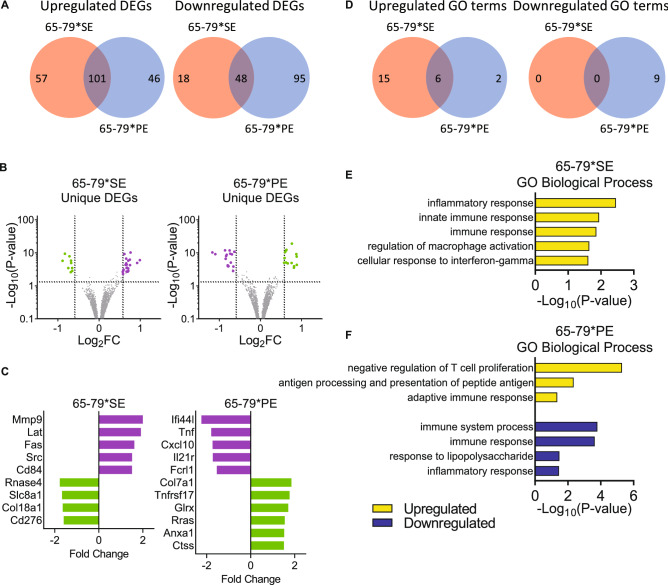
Transcription modulation by 65–79*SE and 65–79*PE under M2 polarizing conditions. Mouse RAW 264.7 macrophages were incubated for 3 days with Il-4 (5 ng/ml) in the presence or absence of 65–79*SE or 65–79*PE and RNA-seq analysis was performed as in Fig. 5. Data are from 6 biological replicates in 2 independent experiments. (**A**) Venn diagrams showing upregulated and downregulated DEGs (P adjusted < 0.05, fold change > 1.5). (**B**) Volcano plots for unique DEGs for 65–79*SE or 65–79*PE. Purple dots denote pro-RA-associated genes; green dots denote RA-protective genes. (**C**) Selected notable genes unique for 65–79*SE or 65–79*PE with known RA-related functions. Purple bars denote pro-RA-associated genes; green bars denote RA-protective genes. (**D**) Venn diagrams of overlapping and unique GO terms derived from upregulated and downregulated DEGs in (**A**). **(E**,**F**) Selected GO Biologic Processes for up- and downregulated DEGs by 65–79*SE (**E**) or 65–79*PE (**F**).

A markedly different transcriptional landscape was observed under M2-polarizing conditions (Fig. 6, Data File S4). Notable amongst the unique upregulated genes by 65–79*PE in M2-polarizing conditions (Fig. 6C) were anti-angiogenic (*Glrx*, *Col7a1*, *Rras*), anti-inflammatory (*Anxa1*, *Rgs2*, *Tnfrsf17*), anti-bone remodeling (*Ctss*), NF-κB inhibitor (*Anxa1*, *Spn*), and activator of the Pi3K-Akt pathway (*Rras*) genes. Downregulated genes in these conditions included many pro-RA genes such as *Tnf*, *Fcrl1*, *Cxcl10*, *Il21r*, *Cxcl2*, *Ifi44l*, *Myo1d*, among others (Fig. 6A–C, Supplemental Table S1, Data File S4A). In spite of the anti-inflammatory effects by IL-4^50,61^, 65–79*SE was able to upregulate various RA and pro-inflammatory genes (e.g. *Adamtsl5*, *Pyr1*, *Mmp9*, *Cd74*, *Cd84* and *Lat*) and downregulate several anti-RA, anti-inflammatory, or pro-M2 genes (e.g. *Rnase4*, *Col18a1Stk17b*, *Cd276*, *Cx3cr1*), although these 65–79*SE effects were weaker than its effect in M1-polarizing conditions (Fig. 6A–C, Supplemental Table S1, Data File S4B). GO analysis (Fig. 6D–F) showed that pathways related to immunity and inflammation were upregulated by 65–79*SE and downregulated by 65–79*PE (Fig. 6E,F, Data Files S4C,D). Thus, under M2 polarizing conditions 65–79*PE and 65–79*SE had reciprocal effects; the former enhanced anti-inflammatory effects, whereas the latter was capable of moderately enhancing an inflammatory transcription profile.

### Upstream regulators

**Figure 7:**
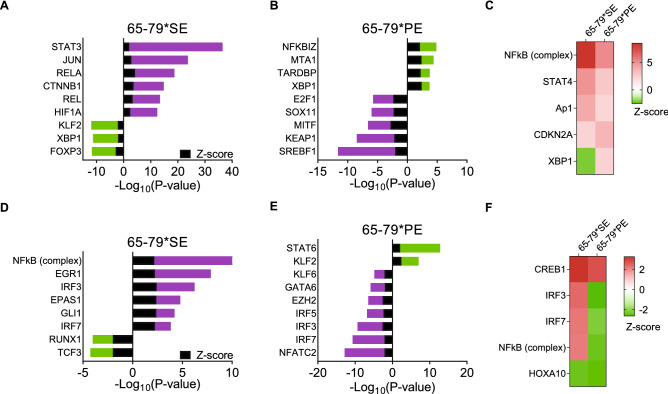
Differential activation of upstream regulators by 65–79*SE and 65–79*PE under M1 or M2 polarization conditions. (**A**,**B**) Predicted activated and inhibited upstream regulators by 65–79*SE (**A**), 65–79*PE (**B**) in M1-polarizing conditions. (**C**) Heatmap of notable upstream regulators for 65–79*SE and 65–79*PE in M1 polarizing conditions. (**D**,**E**) Predicted activated and inhibited upstream regulators by 65–79*SE (**C**), or 65–79*PE (**D**) in M2-polarizing conditions. In (**A**,**B**,**D**,**E**); purple bars denote pro-inflammatory or pro-RA regulators; green bars indicate anti-inflammatory or anti-RA regulators. (**F**) Heatmap of notable upstream regulators for 65–79*SE and 65–79*PE in M2 polarizing conditions. In (**C**,**F**); red denotes predicted activation; green denote predicted inhibition.

To assess the underlying mechanisms driving the gene expression patterns that are induced by 65–79*SE and 65–79*PE, we performed upstream regulator analyses. In M1-polarizing conditions (Fig. 7A–C, Supplemental Table S2, Data File S5A), 65–79*SE was predicted to stimulate activation of pro-inflammatory, pro-RA upstream regulators, such as Stat3, Rel, Rela, Jun, Ctnnb1 and Hif1a, among others, and to inhibit anti-arthritis or anti-inflammatory regulators (e.g. Klf2, Xbp1, Foxp3) (Fig. 7A). In these conditions, 65–79*PE was predicted to activate several anti-inflammatory or NF-κB-inhibiting regulators, such as Nfkbiz, Mta1, Tardbp, Xbp1, and inhibit several key transcription factors known to associate with osteoclastogenesis (Mitf, E2f1), angiogenesis (Srebf1, E2f1, Sox11) and inflammation (Srebf1, Keap1), among others (Fig. 7B, Supplemental Table S2, Data File S5B).

**Figure 8:**
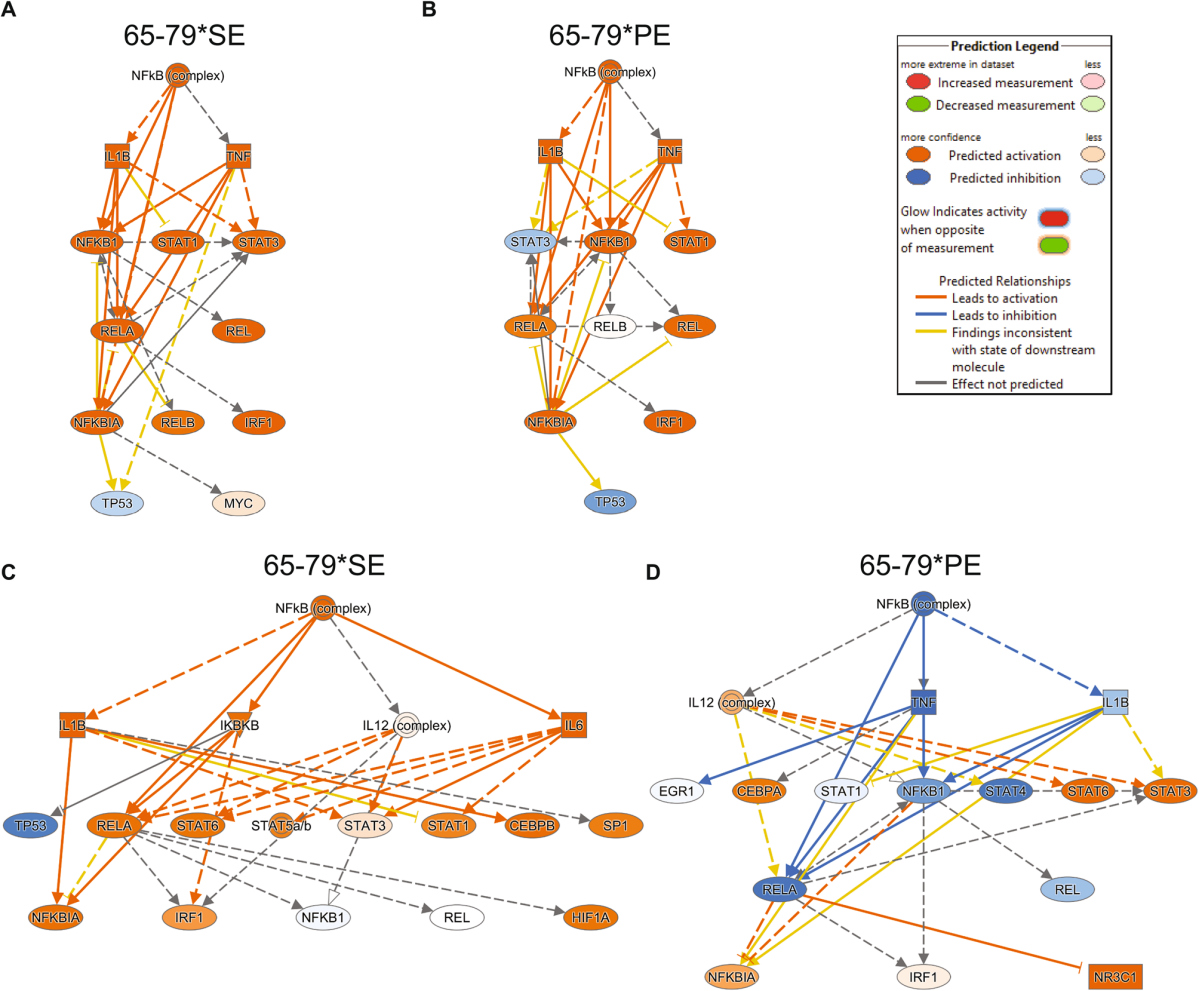
Differential NF-κB pathway activation by 65–79*SE and 65–79*PE under M1 (**A**,**B**) or M2 (**C**,**D**) polarizing conditions. The diagrams were generated through the use of IPA (Qiagen Inc. https://www.qiagenbioinformatics.com/products/ingenuity-pathway-analysis).

Under M2 polarizing conditions (Fig. 7D–F, Supplemental Table S2, Data File S5C,D), 65–79*SE was predicted to exert an activation effect on several pro-inflammatory or pro-angiogenic upstream regulators, including Egr1, Irf3, Irf7, Epas1, Gli1 and NF-κB, and an inhibitory effect on anti-RA, anti-osteoclastogenic or anti-angiogenic upstream regulators, such as Tcf3 and Runx1 (Fig. 7D). Conversely, 65–79*PE was predicted to activate key M2-inducing and anti-arthritis transcription factors Stat6 and Klf2, and inhibit pro-arthritis (Irf5, Irf7), pro-osteoclastogenic (Nfatc2, Ezh2), pro-angiogenic (Nfatc2, Ezh2, Gata6, Klf6, Irf3), pro-inflammatory (Irf5, Klf6), and pro-M1 (Klf6, Irf3, Irf7) upstream regulators (Fig. 7E). Intriguingly, several upstream regulators were predicted to be modulated in diametrically opposite directions by 65–79*SE versus 65–79*PE. For example, in M1-polarizing conditions (Fig. 7C), Xbp1, an anti-inflammatory and NF-κB-inhibiting upstream regulator, was predicted to be inhibited by 65–79*SE, yet activated by 65–79*PE. In M2-polarizing conditions (Fig. 7F), a pro-M1 and pro-angiogenic upstream regulator Irf3, and the pro-M1 pro-arthritis upstream regulator Irf7, and NF-κB were all predicted to be activated by 65–79*SE, and reciprocally inhibited by 65–79*PE. Importantly, consistent with the above findings, and the known role of NF-κB in the pathogenesis of autoimmune diseases, upstream regulators analysis confirmed a pivotal role for NF-κB complex pathway, with opposite outcomes in the presence of 65–79*SE versus 65–79*PE (Fig. 8).

## Discussion

Decades after MHC-restricted AP^2,3^ and HLA-disease association (reviewed in^1^) were independently discovered, it remains unclear to what extent the two processes are mechanistically-coupled. The findings of this study suggest that aside from their nominal role in AP, which is widely considered an underlying mechanism in HLA-disease association, HLA-DR molecules exhibit allele-specific, AP-independent modulatory effects, which might play a role in autoimmune disease pathogenesis as well.

Given the known pivotal role that macrophages play in regulating pro- and anti-inflammatory events, here we focused on these cells. Our findings revealed differential activation of macrophage polarization pathways by *HLA-DRB1* allele-specific gene products. Using primary macrophages derived from two transgenic mouse lines that express distinct human HLA-DRβ molecules, which differ by only 3 amino acid residues in the TAHR, we observed diametrically opposite polarization patterns. Primary macrophages derived from SE Tg (transgenic mice expressing the SE motif 70-QKRAA-74 in the TAHR of the DRβ chain) showed strong predilection to pro-inflammatory M1 macrophage differentiation, while cells derived from PE Tg (expressing a 70-DERAA-74 sequence), displayed preferential differentiation of M2-type macrophages.

The two alleles differentially regulated signaling events. Under M1-polarizing culture conditions, primary macrophages from PE Tg were found to display increased Akt activation and attenuated M1 polarization. In macrophages from SE Tg, on the other hand, SHIP1-mediated Akt inhibition was implicated in enhanced M1 polarization. Under M2-polarizing conditions PE Tg macrophages, but not SE Tg macrophages, selectively enhanced M2 gene expression via the Akt—and possibly Stat6—pathways. Thus, autoimmune disease susceptibility or -protective *HLA-DRB1* alleles were found to reciprocally modulate signaling pathways, which determined the efficiency of pro-inflammatory versus anti-inflammatory macrophage differentiation outcomes.

In vitro*-*differentiated BMDM cultures are devoid of lymphocytes. It is therefore unlikely that the differential macrophage polarization observed here involved AP. To ascertain that AP is indeed not required, and to determine whether the effect could be mapped to the TAHR, we used AP-incompetent 15-mer peptides that correspond to residues 65–79 in the DRβ chain to explore their transcriptional activation in mouse RAW 264.7 and human THP-1 macrophages. The findings revealed that: (A) synthetic peptides corresponding to TAHRs that differ by only 3 amino acid residues activated allele-specific signature transcriptomes; (B) the effect was AP-independent, since it was activated by short, AP-incompetent synthetic peptides; (C) RNA-seq parallels between mouse and human macrophages strengthen the significance of the findings.

Under M1-polarizing conditions, 65–79*SE upregulated the expression levels of many genes that are known to code for pro-inflammatory or known RA disease markers, as well as genes coding for confirmed—or proposed—therapeutic targets, and pathogenic mechanisms, such as osteoclastogenesis, NF-κB activation, angiogenesis, or M1 polarization. Conversely, the SE-expressing TAHR downregulated anti-inflammatory, anti-angiogenesis, inhibitors of NF-κB, and pro-M2 genes. 65–79*PE, on the other hand, upregulated many anti-inflammatory, pro-M2 genes, as well as RA-protective, anti-oxidant, genes. Under M2 polarizing conditions, 65–79*PE downregulated genes and GO processes that are associated with inflammation and RA pathogenesis, while 65–79*SE, notwithstanding the anti-inflammatory tissue culture milieu, managed to upregulate some genes and GO processes known to mediate pro-inflammatory and macrophage activation events.

Importantly, upstream regulator analysis based on gene expression patterns predicted the NF-κB-mediated pathway to be activated by the SE and inhibited by the PE (Fig. 8). This finding is congruent with the key role that NF-κB plays in autoimmune diseases^62–67^. Thus, consistent with their reciprocal impacts on autoimmune disease protection versus susceptibility, the two epitopes dampen (PE) or enhance (SE) pro-inflammatory events.

An RA-protective effect of 70-DERAA-74-coding *HLA-DRB1* alleles has been known for some time^29^, but the mechanism underlying this effect has been unclear. A recent study proposed that antigenic mimicry between vinculin and bacterial proteins presented by HLA-DQ molecules, which are commonly associated with SE-coding *HLA-DRB1* alleles through linkage disequilibrium may be involved in RA^68^. However, PE-coding *HLA-DRB1* alleles have a protective effect in SE-negative individuals as well^69^. Moreover, as discussed above, in addition to their protective effect in RA, PE-coding *HLA-DRB1* alleles have been found to decrease disease risk in many other autoimmune conditions that do not share putative antigens with RA. We propose that allele-specific immune modulatory effects of the kind identified here could be considered as an alternative mechanism in HLA-disease associations that are difficult to explain by AP alone.

It is worth mentioning that based on imputation of genomics data it has been suggested by others that in addition to the 5 residues 70–74 in the TAHR that determine the SE-associated RA disease risk, peptide-binding groove residues 11 and 13 associate significantly with RA risk as well^70^, indirectly suggesting that AP may be involved. However, this imputation-based theory has not yet been experimentally validated. Be that as it may, the mechanism proposed here does not exclude involvement of AP in HLA-associated diseases; it offers explanation to aspects of HLA-disease associations that are inconsistent with AP alone. It is not inconceivable that while presentation of specific antigen(s) may determine the anatomic site(s) involved, the SE and PE polarize the immune response to pro- versus anti-inflammatory modes, thereby shaping the pathogenic outcomes.

Our findings lend support to the MHC Cusp theory, which posits that in addition to their nominal function as antigen presenting receptors, MHC molecules express cusp-region epitopes, which activate evolutionary-conserved, non-MHC receptor-mediated pathways^10^. Whereas cell surface calreticulin has been identified as the SE-interacting receptor^52,54^, the identity of the PE-binding partner is presently unknown. However, the parallels between mouse and human cells observed here suggest that, consistent with the MHC Cusp theory, the PE conceivably activates an evolutionary conserved pathway^10^.

This study has focused on two particular *DRB1* alleles known to reciprocally modulate disease risk in RA. Further research is required to determine if the SE and PE represent a unique case, or whether other HLA molecules express disease-modulating cusp-region epitopes as well.

## Methods

### Mice

Transgenic mice, expressing the human HLA-DR4*04:01 or HLA-DR4*04:02 alleles^43,44^ were kindly provided by Dr. Chela David, at the Mayo Clinic, and are referred to as SE Tg and PE Tg, respectively. The two mouse strains have a mixed (predominantly B6) genetic background and are approximately 99% identical. Both SE Tg and PE Tg express HLA-DR molecules on BMDMs and splenocytes, without statistically significant expression level differences between the two Tg mouse lines (Supplemental Fig. S5). 10–12 week old male mice were housed under specific pathogen-free and temperature-controlled (25 °C) conditions in a 12-h dark/light cycle. All experimental mouse protocols were approved by the University of Michigan Unit for Laboratory Animal Medicine and by the University of Michigan Committee on Use and Care of Animals. All applicable federal, state, local, and institutional laws, regulations, policies, and standards governing animal research were followed. In some experiments, LPS (500 µg/kg) was administered to 10–12 week old male mice using a single i.p. injection. Serum or peritoneal exudate cells (PECs) were collected 4 h. after i.p. injection.

### Reagents

All reagents used, along with vendor names and catalog numbers are listed in Supplemental Table S3.

Synthetic peptides, purchased from Bioworld (Dublin, Ohio, USA) were 95–99% pure. For additional quality assurance, we periodically perform Maldi-TOF Mass Spectrometry and NMR analyses to rule out impurities.

### Primary macrophage culture conditions

Primary mouse BMDMs were isolated and cultured as previously described^56^. Peritoneal macrophages were isolated by injecting ice-cold PBS into the peritoneal cavity. PECs were subsequently collected, centrifuged and washed once with PBS. For qRT-PCR experiments, macrophages were cultured for 3 days in 6-well plates (2 × 10^6^ cells per well) in α-MEM with 10% (v/v) FBS, 100 U/ml penicillin and 100 μg/ml streptomycin, along with 10 ng/ml recombinant macrophage colony stimulating factor (M-CSF). Culture media were refreshed daily. In cell function experiments, instead of M-CSF, macrophages were cultured for 3 days in 20% (v/v) L929 cell conditioned media, in addition to 0.5% (v/v) pyruvate, 10% (v/v) FBS, 100 μg/ml streptomycin and 100 U/ml penicillin. To induce M1 polarization, cells were treated with 1 ng/ml LPS and 20 ng/ml IFNγ for 24 h. To induce M2 polarization, Il-4 and Il-13 (10 ng/ml each) were added for 24 h. For experiments with inhibitors, cells were treated with Ly294004 (5 μM), PF-4708671 (10 μM), or wedelolactone (10 μM) for 1 hr prior to polarization.

### Macrophage cell lines culture conditions

Mouse RAW 264.7 macrophages were maintained in DMEM containing 10% (v/v) FBS, 100 μg/ml streptomycin and 100 U/ml penicillin. Cells were cultured in T75 flasks to confluence and were split every 3 days. THP-1 cells were cultured in a 10% (v/v) FBS-RPMI 1640 medium containing 100 U/ml penicillin and 100 μg/ml streptomycin. To differentiate THP-1 cells into macrophages they were cultured for 3 days with phorbol 12-myristate 13-acetate (PMA, 85 nM). Differentiated cells were then cultured without PMA for an additional 5 days prior to the experiments.

### RNA isolation and qRT-PCR

Mouse cells were lysed with Trizol for RNA isolation. Direct-Zol™ RNA miniprep (Zymo research) was used to isolate total RNA. Isolation of RNA from THP-1 cells was carried out with a RNeasy Plus Mini kit (Qiagen). The High-Capacity cDNA Reverse Transcription Kit (Applied Biosystems) was used to synthesize cDNA. qRT-PCR was performed by Fast SYBRTM Green Master Mix (Applied Biosystems), with sets of primers as listed in Supplementary Table S4, using a StepOnePlus Real-Time PCR system (Applied Biosystems). A StepOne Software was used to analyze the data with the ΔΔCT method.

### Immunoblots

After being washed with ice-cold PBS, cells were lysed in RIPA buffer (Sigma) with EDTA-free protease phosphatase inhibitors (Roche Diagnostics). Protein concentration in lysates was determined using the DC protein assay (BioRad). Proteins were loaded onto 4–20% SDS–PAGE gels (Invitrogen), and after electrophoretic separation, transferred to nitrocellulose membranes (BioRad), followed by incubation with appropriate primary and secondary antibodies. All primary antibodies were diluted 1:1000 in 5% (v/v) BSA (Sigma-Aldrich), except β-Actin (1:4,000). The primary antibodies used were anti: Akt (#9272), pAkt (S473, #9271), STAT6 (#9362), pSTAT6 (Y641, #56554), SHIP1 (#2728), pSHIP1 (Y1020, #3941), PTEN (#9559), pPTEN (S380/T382/383, #9549) (all from Cell Signaling Technology), or anti-β-Actin (Invitrogen, BA3R). Second-stage antibodies included Anti-Rabbit IgG HRP-conjugated (Cell Signaling Technology, 1:1000) or anti-mouse IgG HRP-conjugated (GE Healthcare, 1:8000). All blots, except those in Supplementary Fig. S1B, were stripped and re-hybridized as one piece. In Supplementary Fig. S1B, membranes were first hybridized with an anti-phosho-SHIP1 antibody, then stripped, blocked and the top part was cut and hybridized with anti-total SHIP1 antibody, while the bottom part was hybridized with anti-β-Actin antibody. Band visualization was performed using a SuperSignal West Pico Plus ECL substrate (Thermo Scientific) and an Omega Lum C imaging system (Gel Company). Band quantification was performed in duplicates with the ImageJ software.

### Cytokine measurements

Tnfα, Il-12p70, Il-10 and Il-6 were quantified in culture supernatants by ELISA kits (R&D). Serum cytokine levels were measured using Quantibody Mouse Cytokine array 1 (RayBiotech). Slides were scanned by RayBiotech Service Department and data were analyzed using a RayBiotech Mouse Cytokine Array 1 software (QAM-CYT-1-SW). Sample concentrations were derived from mean fluorescence intensities, relative to standard curves, generated using the manufacturer’s standards.

### NO and arginase assays

NO production was quantified using the fluorescent NO dye 4,5-diaminofluorescein diacetate (DAF-2DA) as previously described^71^. Cells were first plated overnight in flat‐bottom, 96‐well plates, then washed with DMEM/phenol red-free medium (Sigma). Cultures were then loaded with 20 μM DAF‐2DA at 37 °C for 1 h. in the dark. Fluorescence levels were recorded every 5 min over a period of 500 min using a Synergy H1 hybrid reader system (Biotek) at excitation/emission wavelengths of 488/515 nm. NO production rates are expressed as fluorescence units (FU) per minute. Measurement of arginase activity was carried out using the Arginase Activity Colorimetric Assay Kit (Biovision).

### RNA-Seq

Cells were incubated with or without IFNγ (5 ng/ml) or Il-4 (5 ng/ml) in the presence or absence of 15-mer peptides 65–79*SE or 65–79*PE (100 μg/ml) and medium was refreshed at 48 h. At 72 h., total RNA was isolated using the Direct-Zol™ RNA miniprep (Zymo research). Genomic DNA was removed using the Turbo DNA-Free kit (Ambion). Total RNA concentration and integrity were determined with an Agilent Bioanalyzer (Agilent). All RNA samples had an integrity number of 7.5 or higher. Two hundred ng of total RNA were used to generate libraries using the TruSeq Stranded mRNA Sample Preparation Kit (Illumina). Single-end reads of 100 bp for each sample were produced with Illumina’s HiSeq2500v4 instrument. Raw counts were attained using featureCounts from the Rsubread1.5.0p3 package and Gencode-M12 gene annotations using only uniquely aligned reads. For THP-1, Raw counts were attained using featureCounts from the Rsubread-1.6.1 package and -Gencode28-hg38 gene annotations using only uniquely aligned reads. DESeq2-1.16.1 within R-3.4.1 was used to perform data normalization and differential expression analysis with an adjusted p value threshold of 0.05. Mouse annotations were verified using the MGI database (http://www.informatics.jax.org/index.shtml)^72^ and only protein encoding genes were considered. Genes with a fold-change of > 1.5 and p values of 0.05 were used for GO term analysis. RA relevant genes were identified based on published literature. The DAVID bioinformatics database (version 6.8)^73^ was used for GO analysis, using an Expression Analysis Systematic Explorer (EASE) score threshold of 0.1 for detection of gene enrichment. GO terms with an FDR of 5% were considered significant. To identify unique GO terms, significant GO terms obtained in 65–79*SE-stimulated cells in either M1- or M2-polarizing culture conditions were compared to those obtained in 65–79*PE-stimulated cells under the same polarizing culture conditions.

### Upstream regulator analysis

Ingenuity Pathway Analysis (QIAGEN Inc., CA, www.ingenuity.com/index.html) was used to perform upstream regulator analysis. Genes differentially expressed in the presence of 65–79*SE or 65–79*PE were analyzed to identify transcriptional regulators that may be responsible for the gene expression changes observed. Only transcriptional regulator relationships observed using experimental data were used in this analysis. A z score was used to examine the robustness of predicted activation or inhibition of specific transcription regulators based on enrichment and differential expression (upregulation or downregulation) in the presence of 65–79*SE or 65–79*PE. *p* values < 0.05 by Fisher’s Exact Test after correction for multiple testing were considered significant.

### Flow cytometry

BMDMs were grown in αMEM with M-CSF (10 ng/ml) for 4 days. Splenocytes were isolated as single-cell suspensions by mashing spleen tissues through a 70 µm cell strainer. Red blood cells were lysed using an RBC lysis buffer (Biolegend) according the manufacturer’s instructions. Non-permeabilized BMDMs or splenocytes were incubated for 30 min on ice with a phycoerythrin-conjugated anti-HLA-DR antibody (Clone L243, Biolegend) and Ghost Dye Violet 510 (Cat# 13-0870-T100, Tonbo). Cells were analyzed using a ZE5 flow cytometer (Bio-Rad). Histograms were generated using the FlowJo software, version 10.7.1.

### Statistics

Data are shown as the mean and SEM calculated using a GraphPad Prism Software (version 7). Unless otherwise specified, a 2-way ANOVA was used to determine significance. *p* values < 0.05 were considered significant. Bar graphs were generated using GraphPad Prism version 7.0.0 from GraphPad Software, CA, www.graphpad.com.

## Supplementary Information

Supplementary Information 1.

Supplementary Information 2.

Supplementary Information 3.

Supplementary Information 4.

Supplementary Information 5.

Supplementary Information 6.

## Data Availability

The RNA-seq data have been deposited in NCBI's Gene Expression Omnibus and are accessible through GEO accession number GSE159821.
