# Supplementary material for: HLA-DRB1 allelic epitopes that associate with autoimmune disease risk or protection activate reciprocal macrophage polarization
Source: Sci Rep. 2021 Jan 28;11:2599. doi: 10.1038/s41598-021-82195-3 (PMC7844024; doi:10.1038/s41598-021-82195-3)
Supplement: Supplementary file 6 — Supplementary Information 6. [file 41598_2021_82195_MOESM6_ESM.pdf]

Supplementary Materials for

HLA-DRB1 Allelic Epitopes that Associate with Autoimmune Disease Risk or Protection Activate Reciprocal Macrophage Polarization

Vincent van Drongelen[1], Bruna Miglioranza Scavuzzi[1], Sarah Veloso Nogueira[1], Frederick W. Miller[2] Amr H. Sawalha[1,3], Joseph Holoshitz[1]

[1] Department of Internal Medicine, University of Michigan, Ann Arbor, MI, 48109, USA.

[2] Environmental Autoimmunity Group, National Institute of Environmental Health Sciences, Research Triangle Park, NC, 27709, USA.

[3] Current address: Departments of Pediatrics and Internal Medicine, University of Pittsburgh, Pittsburgh, PA 15224, USA.

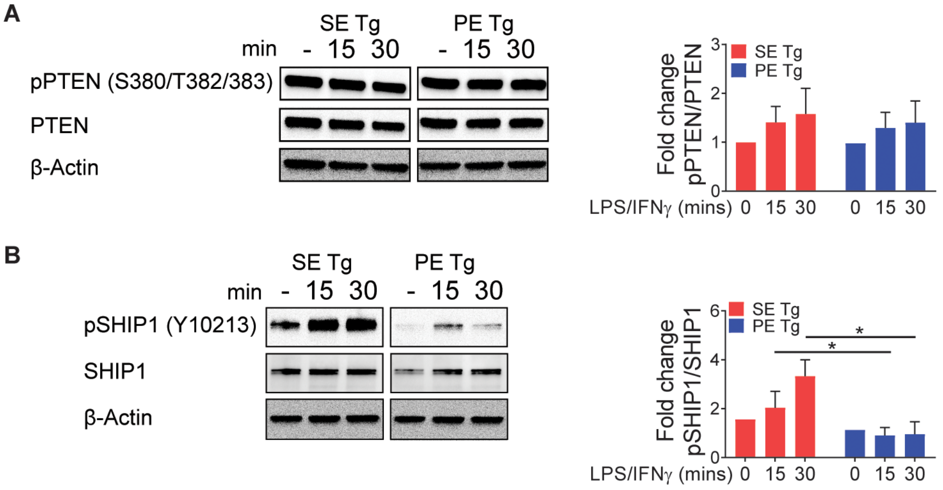

Supplemental Fig. S1. Role of Pi3K regulating phosphatases

(**A**) Immunoblot for pPTEN (Ser380/Thr382/383) and PTEN under M1 polarizing conditions [LPS (1 ng/ml) + IFNγ (20 ng/ml)] for 15-30 min.

(**B**) Immunoblot for pSHIP1 (Tyr1021) and SHIP1 under M1 polarizing conditions as in (A). Quantification data represent mean + SEM of 3 independent experiments. 2-way ANOVA, *$p<0.05$

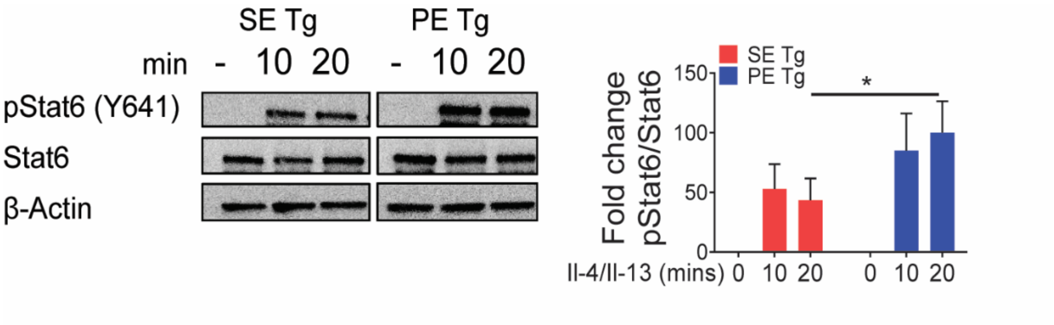

Supplemental Fig. S2. Differential Stat6 phosphorylation in SE Tg and PE Tg BMDMs under M2 polarizing conditions

Western blot of pStat6 (Tyr641) and Stat6 in BMDMs derived from SE Tg or PE Tg mice following treatment with IL-4 (10 ng/ml) and IL-13 (10 ng/ml) for 10 or 20 minutes. Data on the right represent mean and SEM of 3 independent experiments. 2-way ANOVA, *P<0.05.

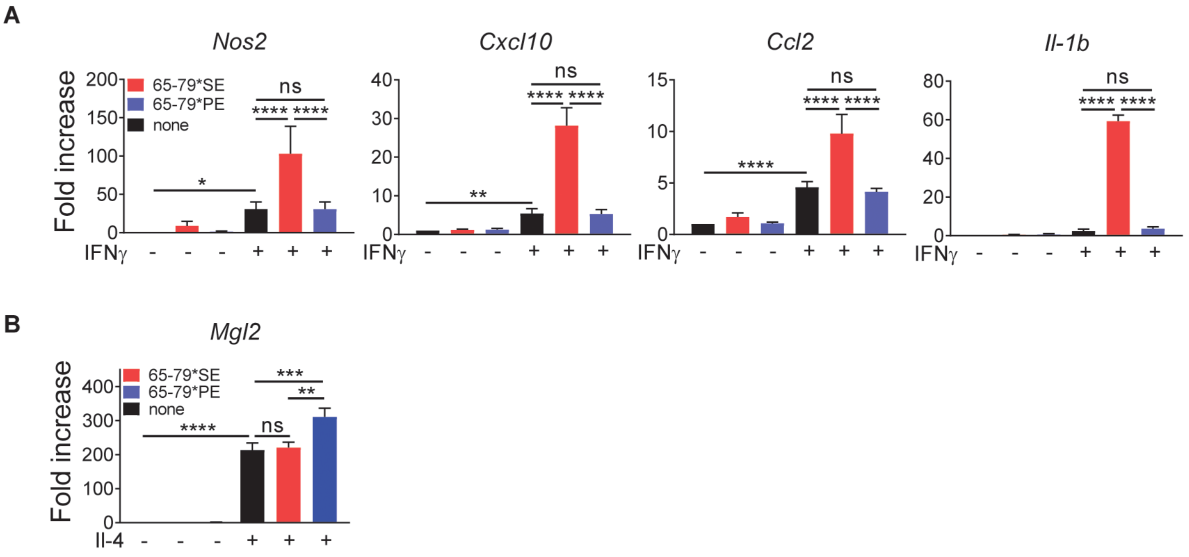

Supplemental Fig. S3. **Differential transcriptional response to 65-79*SE or 65-79*PE under M1 and M2 polarizing conditions**

Mouse RAW 264.7 macrophages were incubated for 3 days with IFNγ (5 ng/ml) or IL-4 (5 ng/ml) in the presence or absence of 100 µg/ml 65-79*SE or 65-79*PE.

(**A**) qRT-PCR for the M1 genes *Nos2*, *Cxcl10*, *Ccl2* and *Il-1b*. Data represent mean + SEM of 3 biological replicates.

(**B**) qPCR for M2 gene *Mgl2*. Data represents mean + SEM of 5 biological replicates, 1-way ANOVA, *P<0.05, **P<0.01, ****P<0.0001.

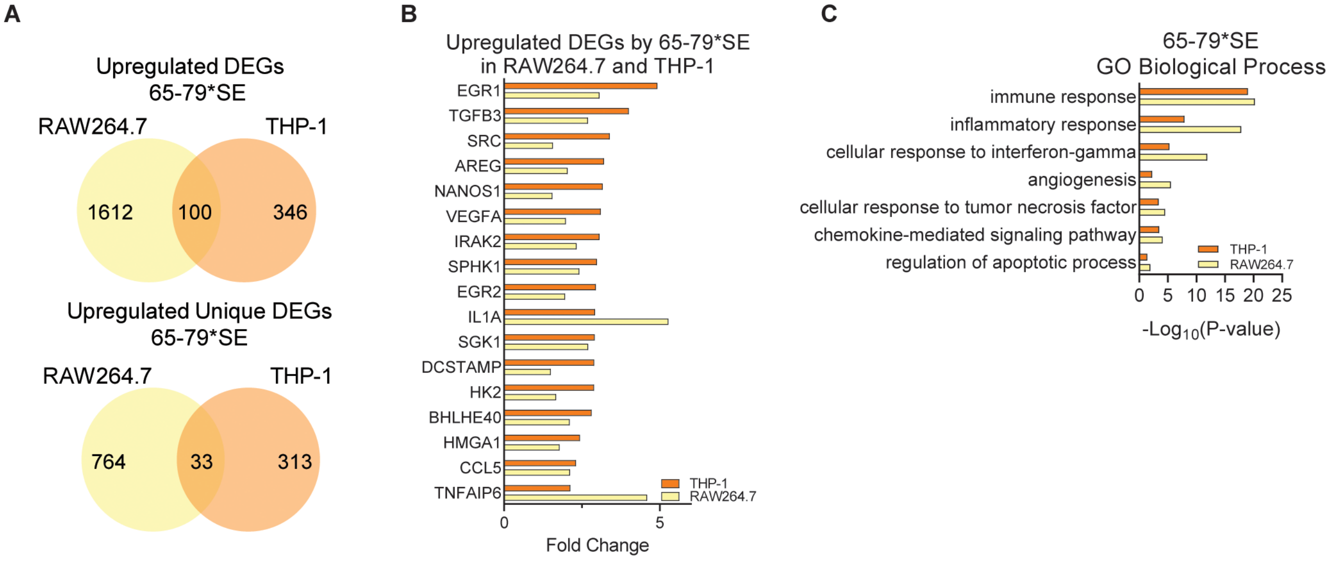

D

	THP-1		RAW264.7	
Term	Benjamini	Padj	Benjamini	Padj
hsa05323:Rheumatoid arthritis	5.06E-17	5.06E-19	2.94E-07	2.94E-09
hsa05332:Graft-versus-host disease	2.51E-14	2.51E-16	2.08E-13	2.08E-15
hsa04940:Type I diabetes mellitus	2.01E-12	2.01E-14	2.41E-12	2.41E-14
hsa04612:Antigen processing and presentation	2.74E-12	2.74E-14	1.68E-13	1.68E-15
hsa05330:Allograft rejection	5.89E-11	5.89E-13	6.24E-12	6.24E-14
hsa05416:Viral myocarditis	2.32E-10	2.32E-12	7.81E-09	7.81E-11
hsa05320:Autoimmune thyroid disease	8.13E-09	8.13E-11	1.47E-08	1.47E-10
hsa05140:Leishmaniasis	6.46E-08	6.46E-10	3.42E-08	3.42E-10
hsa05152:Tuberculosis	7.57E-08	7.57E-10	3.08E-08	3.08E-10
hsa05321:Inflammatory bowel disease (IBD)	1.11E-07	1.11E-09	3.63E-08	3.63E-10
hsa04514:Cell adhesion molecules (CAMs)	9.81E-07	9.81E-09	7.41E-10	7.41E-12
hsa05150:Staphylococcus aureus infection	1.00E-06	1.00E-08	8.89E-14	8.89E-16
hsa04145:Phagosome	2.03E-06	2.03E-08	6.40E-12	6.40E-14
hsa05168:Herpes simplex infection	2.22E-06	2.22E-08	2.21E-12	2.21E-14
hsa05310:Asthma	2.40E-06	2.40E-08	1.82E-04	1.82E-06
hsa05166:HTLV-I infection	9.53E-06	9.53E-08	1.77E-12	1.77E-14
hsa04060:Cytokine-cytokine receptor interaction	1.35E-05	1.35E-07	8.24E-12	8.24E-14
hsa05145:Toxoplasmosis	1.36E-05	1.36E-07	1.93E-08	1.93E-10
hsa04672:Intestinal immune network for IgA production	1.37E-05	1.37E-07	6.94E-08	6.94E-10
hsa05164:Influenza A	8.04E-04	8.04E-06	1.49E-08	1.49E-10
hsa05169:Epstein-Barr virus infection	8.26E-04	8.26E-06	2.61E-05	2.61E-07
hsa04668:TNF signaling pathway	9.07E-04	9.07E-06	2.55E-05	2.55E-07

Supplemental Fig. S4. Corresponding upregulated DEGs between THP-1 and RAW 264.7 cells under M1 polarizing conditions

THP-1 (human) or RAW264.7 (mouse) macrophages were incubated for 3 days with IFNγ (5ng/ml) in the presence or absence of 100 µg/ml 65-79*SE and RNA-seq analysis was performed on isolated RNA. THP-1 data are from 4 independent experiments. RAW 264.7 data are from 6 biological replicates in 2 independent experiments.

(**A**) Venn diagrams showing the overlap between THP-1 and RAW 264.7 cells for all and unique upregulated DEGs (P adjusted <0.05, fold change >1.5).

(**B**) Notable unique genes with RA relevance upregulated by 65-79*SE in both THP-1 and RAW 264.7 cells.

(**C**) Notable GO Biologic Processes unique for 65-79*SE for upregulated DEGs in both THP-1 and RAW 264.7 cells.

(**D**) KEGG pathways unique for 65-79*SE for upregulated DEGs in both THP-1 and RAW 264.7 cells.

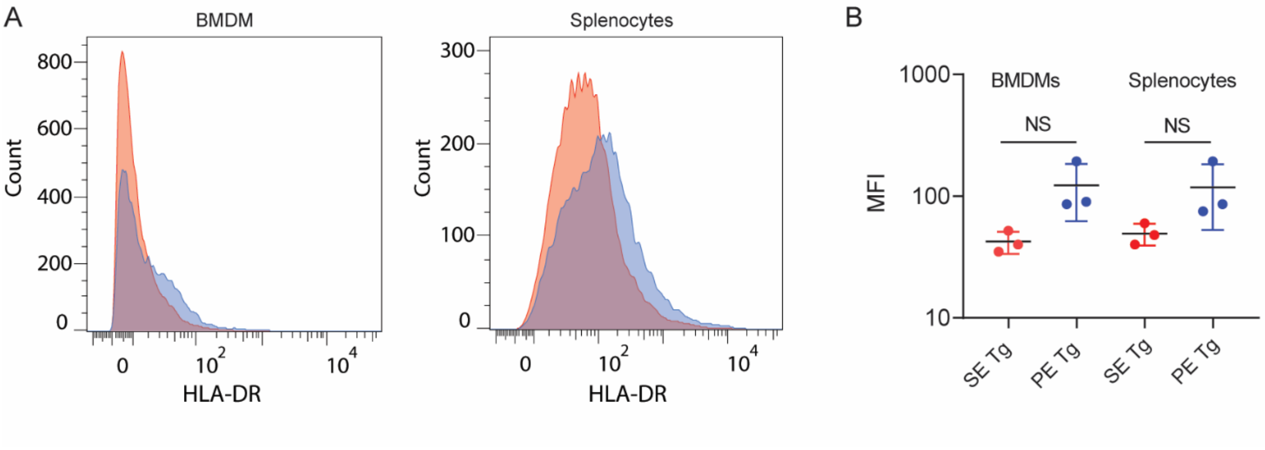

Supplemental Fig. S5. HLA-DR surface expression in SE Tg and PE Tg mice
(**A**) Representative flow cytometry histograms of SE Tg- and PE Tg-derived BMDMs (left) or splenocytes (right), stained for HLA-DR surface expression. Red histograms represent SE Tg, blue histograms represent PE Tg.
(**B**) Median fluorescence intensity (MFI) for HLA-DR in SE Tg- and PE Tg-derived BMDMs and splenocytes. Error bars represent SD; n = 3 in all cell types. Determination of statistical significance for each cell type, performed by 2-tailed Student *t*-test found no significance (NS). The transgenic genotype among the 4 group had no statistically significant effect, as determined by one-way ANOVA. Hitograms were generated using FlowJo Software (version 10.7.1.), Ashland, OR, www.flowjo.com

Supplemental Table S1: DEGs with RA relevance

S1A. Notable unique DEGs modulated by 65-79*SE under M1 polarizing conditions

Upregulated			
	**Fold Change**	**Padj**	**RA-relevant roles/functions**
Dusp4	3.9137	1.61E-52	Pro-angiogenic;<br>Pro-Th17 polarization
Vav1	1.6236	1.44E-51	RA-associated;<br>Neutrophil activation;<br>Involved in CD28-mediated T cell activation through NF-kB pathway
Cd44	2.3622	1.95E-47	MIF co-receptor; plays a role in RA pathogenesis<br>Marks an RA synovial tissue enriched peripheral T helper cell;<br>Marker of effector helper memory T cell
Kpna4	3.1673	4.00E-43	Involved in NF-kB nuclear translocation
Mdm2	2.4483	9.82E-41	Pro-arthritis in RA and CIA
Stat3	1.7648	2.93E-36	Important in RA pathogenesis;<br>Possible treatment target;<br>Interaction with HIF1a
Pgf	13.4457	5.96E-34	Pro-RA;<br>Pro-angiogenesis
Atf4	1.6959	9.85E-34	Pro-osteoclastogenic
Irak2	2.3285	3.83E-30	Genetic risk factor in RA;<br>Pro-inflammatory
Hmga1	1.7851	5.00E-30	Pro-angiogenesis;<br>Pro-inflammatory
Ptpn2	1.8440	7.19E-29	RA- and JIA associated gene
Cd83	3.1311	5.70E-28	RA-associated
Adam8	2.5072	2.71E-27	Pro-osteoclastic;<br>Pro-inflammatory;<br>Pro-angiogenic
Jak1	1.8108	2.79E-25	Therapeutically targeted RA signaling pathway
Sgk1	2.7032	4.77E-23	Pro-Th17
Il7r	2.1721	1.84E-21	Pro-inflammatory B cell
Relb	1.8324	4.39E-18	NF-kB factor associated with RA
Egr1	3.0682	4.51E-18	Pro-angiogenic
Runx1	1.6525	6.03E-18	Th17-polarizing
Bhlhe40	2.1067	1.91E-16	Pro-angiogenic;<br>Pro-inflammatory
Vegfc	2.3484	2.44E-15	RA and angiogenesis associated
Traf1	2.1511	5.46E-15	RA risk locus
Il1a	5.2812	8.42E-15	Pro-osteoclastogenic;<br>Therapeutically targetable in RA
Nxn	2.9583	3.77E-13	Pro-inflammatory
Vegfa	1.9826	7.44E-13	Angiogenesis, RA;<br>Angiogenesis and osteoclast-dependent bone remodeling;<br>Pro-osteoclastogenic
Ccr1	2.2640	2.58E-12	Overexpressed in CIA joint;<br>Overexpressed in RA;<br>Potentially targetable in RA
Tnfaip3	2.2224	3.27E-12	RA risk locus
Jak3	1.8332	4.58E-12	A pathogenic enzyme in RA;

			Therapeutically targeted by tofacitinib
Src	1.5709	1.71E-11	Mediates IL-6 production in synovial fibroblasts;<br>Pro-osteoclast; Anti-osteoblast
Klf6	1.7604	2.37E-11	Pro-M1 polarization
Cxcr4	2.4499	1.19E-09	Involved in RA and angiogenesis;<br>Therapeutically targetable
Hk2	1.6686	1.25E-09	Specifically expressed in RA and a proposed therapeutic target;<br>Pro-Th17; Pro-arthritis; Pro-angiogenic
Hbegf	3.7274	3.54E-09	IL-17-activated;<br>Pro-angiogenic
Smad6	2.1062	1.16E-08	Pro-angiogenic
Gata3	1.8774	2.52E-08	Pro-angiogenic
Dcstamp	1.5013	5.67E-08	Pro-osteoclastogenic
Vim	1.7084	4.29E-07	Considered an autoantigen in RA
Tnfaip6	4.5943	4.24E-06	Pro-angiogenic
Mmp14	3.2325	4.38E-06	Expressed in RA synovium;<br>Plays a role in joint destruction;<br>Therapeutically targetable
Olr1	4.0542	1.29E-05	Pro-angiogenic
Tgfb3	2.6955	3.02E-05	Pro-angiogenic
Mmp12	2.7932	4.31E-05	Pathogenic role in inflammatory arthritis
Mmp13	2.9020	3.34E-04	Overexpressed;<br>Plays a role in joint destruction
Cxcr1	3.0881	4.71E-04	Transduces IL-8 pro-osteoclastogenic signal;<br>Pro-angiogenic
Egr2	1.9622	5.10E-04	Risk factor for RA;<br>Upregulated by TNF
Sphk1	2.4153	3.65E-03	Over-expressed in RA;<br>Pro-FLS activation;<br>Pro-angiogenic;<br>Proposed therapeutic target;<br>Downstream enzyme in the TNFa pathway
Areg	2.0454	7.52E-03	Over-expressed in RA synovium; Up-regulated by IL-1b;<br>Stimulates SFL in RA
Nanos1	1.5502	1.03E-02	Increased expression in CIA;<br>Pro-angiogenic;<br>Pro-M1 polarization;<br>Pro-inflammatory

**Downregulated**			
	**Fold Change**	**Padj**	**RA-relevant roles/functions**
Abcb1b	1.7458	3.41E-38	Decreased levels in experimental arthritis rats
Akap1	1.6996	1.66E-50	Ant-inflammatory;<br>Anti-hypoxia-induced stress
Anxa3	1.7587	1.61E-33	RA susceptibility locus
Arhgap30	1.5626	3.90E-56	Inhibitor of Wnt/β-catenin pathway
Atp2a3	2.1019	3.98E-41	Its inhibition leads to NF-kB p65 translocation and improved survival of CD4 T cells
Bag2	1.7373	1.42E-26	Repressed by NF-kB
Casp9	1.7946	2.62E-27	Pro-apoptotic; anti-inflammatory
Cd81	1.6682	2.86E-24	Pro-Th2 differentiation;<br>Anti-angiogenic

Chd4	1.5142	4.45E-29	Mi-2. A co-repressor of T, B and plasma cell development
Ctdsp1	1.7170	1.81E-36	Anti-angiogenic
Cyp51	2.2615	1.50E-41	Anti-inflammatory - KO leads to inflammation
Dock2	1.5969	3.02E-64	Immune regulatory
Fads1	1.6000	4.19E-77	RA risk association;<br>Supporting M2 differentiation
Fkbp11	3.1032	1.17E-19	Anti-ER stress and inflammation;<br>Pro-osteogenic
Glo1	1.7115	1.24E-29	Part of HLA extended haplotype;<br>Anti-oxidant: downstream of Nrf2
Hsd17b4	1.8641	1.31E-75	Anti-inflammatory
Ikbip	2.0823	6.82E-27	Pro-apoptotic
Klf2	1.8713	2.40E-10	Pro-M2;<br>Anti-angiogenic;<br>Anti-inflammatory
Lpcat3	1.9358	1.34E-36	Pro-M2 polarization;<br>Anti-inflammatory
Lyl1	1.9867	2.95E-26	Anti-angiogenic
Mapk14	1.6445	8.34E-30	Anti-angiogenesis
Parp1	1.5292	9.48E-45	Anti-osteoclastogenic
Patz1	1.8747	1.11E-27	Anti-angiogenesis;<br>Inhibiting NF-kB pathway
Pi16	2.7190	2.22E-10	Inhibits MMP2;<br>Treg subset marker
Pon2	1.9140	1.45E-35	Pro-M2;<br>Anti-inflammatory;<br>Anti-oxidant;<br>Anti-atherogenic
Qdpr	1.6821	4.49E-27	Anti-oxidative damage
Scarb2	1.7086	2.80E-31	CD36;<br>Anti-inflammatory
Slc13a3	2.4445	2.05E-09	Anti-oxidative stress
Sub1	1.9104	4.56E-32	Anti-oxidative stress
Txndc5	2.1280	1.26E-33	Anti-oxidative stress
Ywhab	1.6189	5.95E-61	Anti-angiogenic; Associated with resolution of inflammation and M2 differentiation

S1B. Notable unique DEGs modulated by 65-79*PE under M1 polarizing conditions

**Upregulated**			
	**Fold Change**	**Padj**	**RA-relevant roles/functions**
Acox2	2.3960	1.46E-03	Anti-oxidative stress
Alkbh2	1.5265	1.56E-12	Post-inflammatory DNA damage-repairing enzyme
Bglap	3.0692	7.84E-05	Pro-osteoblast
Cdsn	2.9308	2.04E-06	Anti-IL-1b, anti-inflammatory
Def6	1.6748	2.58E-21	Anti-osteoclastogenic;<br>Limits proliferation of TFH cells in mice via alteration of mTORC1 signaling
Dock3	2.6558	6.72E-07	Anti-oxidative stress
Fbxl12	1.7321	1.01E-10	pro-osteoblast
Filip1l	1.8170	4.62E-15	Anti-angiogenesis
Fyn	1.6248	5.26E-13	Anti-angiogenesis;<br>Anti-ER stress

Gpr65	1.7215	1.90E-14	Anti-osteoclastogenic
Havcr2	1.7619	3.13E-21	Pro-immune tolerance;<br>Anti-RA, inversely correlated with disease activity
Htatip2	1.6956	2.52E-12	Anti-angiogenesis
Il17rc	1.7079	6.22E-16	Anti-IL-17<br>(in a soluble IL-17RC form)
Inpp4b	2.5990	1.09E-03	Anti-angiogenic;<br>Anti-osteoclast
Klhl22	1.6139	1.20E-30	Activates mTORc
Mfsd2a	2.1315	6.36E-07	Anti-inflammatory;<br>Anti-angiogenic
Mt2	1.8269	1.44E-23	Anti-oxidant;<br>Anti-inflammatory;<br>Inhibits NF-kB activation
Nrf1	1.5208	1.11E-10	Anti-inflammatory;<br>Anti-m1 polarization
Ogg1	1.6344	1.51E-13	Anti-inflammatory
Osgin1	1.6636	2.40E-11	Cyto-protective, Nrf2-transcriptional target
Pdcd2	1.7995	4.16E-24	Inhibits TNFa
Pycr1	1.8976	7.72E-11	Anti-oxidative stress
Stc2	1.9779	1.70E-11	Anti-ER and oxidative stress
Stk10	1.6032	1.14E-10	Anti-inflammatory;<br>Anti-lymphocyte activation and inflammation
Themis2	1.8008	3.61E-22	Inhibits macrophage activation
Tlr1	1.5914	6.78E-17	Pro-M2
Traf3ip3	1.8829	1.29E-25	Regulator of Treg;<br>Interacts with mTORC1

**Downregulated**			
	**Fold Change**	**Padj**	**RA-relevant roles/functions**
Atp6v0d2	-2.7543	1.49E-48	Pro-osteoclastogenic
Oas2	-2.6083	2.59E-40	RA signature gene regulating immune response;<br>Innate, type 1 IFN-induced
Dnmt3a	-2.0215	7.63E-35	Pro-osteoclastogenic;<br>Over-expressed in RA synovium;<br>Expression in macrophages directly correlates with pro-inflammatory and inversely with anti-inflammatory state
St3gal1	-2.7146	5.71E-33	Pro-angiogenic
Syk	-2.1744	3.26E-31	Pro-arthritogenic in RA model;<br>Therapeutic target in RA;<br>Pro-osteoclastogenic
Colec12	-2.5837	1.16E-29	Pro-inflammatory
Glul	-2.0115	3.84E-27	Pro-angiogenic
Epn2	-1.9301	6.75E-27	Pro-angiogenic
Ckb	-2.1008	2.91E-26	Pro-osteoclastogenic
Sh3kbp1	-2.0065	6.88E-25	NF-kB activation;<br>B and T cell immune response
Ddx58	-1.8876	1.69E-23	Innate immune system;<br>Activator of NF-kB and IRF-3;<br>Abundant in RA synovial fibroblasts
Jun	-2.8318	4.77E-23	Increased expression in activated synovial cells and in patients;<br>Pro-arthritis in mice
Gtf2i	-1.5113	3.26E-22	RA-associated locus in Asians;<br>Autoimmunity-associated locus

Tnfrsf9	-4.5323	4.43E-22	Released by activated lymphocytes and is abundant in RA;<br>Therapeutically targetable in arthritic mice; RA severity locus in African Americans
Tspan5	-1.6426	1.19E-21	Pro-osteoclastic
Lrp1	-2.2938	1.43E-21	Co-receptor of SE ligand 65-79*0401
Bhlhe41	-1.9412	1.52E-20	Abundant in RA synovium, increases IL1b
F10	-5.0286	4.06E-20	Factor X. Potential key role in RA progression
F7	-4.0622	6.75E-09	Factor VII. Plays a role in the pathogenesis of chronic destructive arthritis in RA; NF-Kb activator and IL-8 inducer; Inducer of VEGF in fibroblasts; Pro-angiogenic
Arhgap24	-3.0636	1.17E-07	Rac1. Plays a role in RA synoviocyte activation;<br>Pro-arthritogenic in mice;<br>Pro-angiogenic
Sulf2	-3.0302	4.16E-07	Expressed in RA synoviocytes and involved in their activation state; Proangiogenic
Acp5	-2.4707	6.03E-07	Tartrate resistant acid phosphatase - a key plyer in osteoclast-mediated bone erosion
Scg2	-3.2210	8.12E-07	Pro-angiogenic
Fabp4	-2.7021	2.42E-06	Increased levels in RA;<br>Pro-angiogenic
Adamts7	-2.5569	4.48E-04	Over-expressed in RA joint tissues and degrades cartilage;<br>Arthritogenic in mice
Src	-1.5104	2.94E-09	Pro-osteoclast

S1C. Notable unique DEGs modulated by 65-79*SE under M2 polarizing conditions

**Upregulated**			
	**Fold Change**	**Padj**	**RA-relevant roles/functions**
Slc31a2	1.7121	1.09E-10	Pro-oxidative
Adamtsl5	1.5423	1.07E-09	Inducer of IL-17
Ryr1	1.5904	3.06E-07	Pro-oxidative;<br>NF-kB inducible
Layn	1.6915	8.93E-07	Enhances inflammation and cartilage destruction and secretion of IL-8
Mmp9	2.0024	1.43E-06	RA;<br>Pro-osteoclast;<br>Pro-angiogenesis
Trpc4	1.6466	2.31E-06	Pro-angiogenic
Lat	1.9123	1.56E-05	RA
Atf3	1.5921	2.14E-05	Pro-angiogenic
Il11ra1	1.6034	5.37E-05	Pro-osteoclast and bone remodeling
Ahnak2	1.5733	6.13E-05	Associated with RA risk
Lcn2	1.6957	7.38E-05	Associated with RA
Fas	1.6118	3.50E-04	Associated with RA
Anpep	1.6007	4.24E-04	CD13
Pgf	1.6470	5.30E-04	Pro-RA;<br>Pro-angiogenesis
Arg2	1.5223	1.00E-03	M1 macrophage marker;<br>Correlates with RA disease activity;<br>Pro-oxidative
Src	1.5127	1.45E-03	Pro-osteoclast
Ccl7	1.6488	1.78E-03	Activates monocyte migration in RA

Acod1	1.5991	2.33E-03	Pro-inflammatory;<br>M1 macrophage marker
Atp6v0d2	1.6287	2.43E-03	Pro-osteoclastogenic
Cd74	1.5001	2.64E-03	MIF in RA
Cd84	1.5074	5.95E-03	RA risk factor and disease marker

**Downregulated**			
	**Fold Change**	**Padj**	**RA-relevant roles/functions**
Rnase4	1.7690	6.96E-10	Regulator of innate immune response
Col18a1	1.6365	2.18E-08	Anti-angiogenesis;<br>Proposed treatment for RA
Stk17b	1.5931	2.27E-06	Anti-memory T cell development; Anti-T cell activation
Zfp608	1.8395	2.59E-06	Anti-thymocytes - prevents RAG1 and RAG2
Cd276	1.5893	1.60E-05	M2-polarizing;<br>Inhibitor of T cell response
Txnip	1.5641	4.18E-04	Inhibitor of HIF1a
Slc8a1	1.6686	4.27E-04	Inhibitor of osteoclast-mediated bone resorption
Nt5e	1.5624	1.74E-03	Protective against CIA;<br>promotes M2;<br>Marker of Treg and mediates immune suppression
Cx3cr1	1.6004	2.95E-03	M2 marker in mice;<br>Required for Treg proliferation and IL-10 production

S1D. Notable unique DEGs modulated by 65-79*PE under M2 polarizing conditions

**Upregulated**			
	**Fold Change**	**Padj**	**RA-relevant roles/functions**
Glrx	1.7104	2.88E-19	Anti-redox; anti-angiogenesis
Anxa6	1.5611	9.59E-13	NF-kB modulator
Car5b	1.5519	3.09E-12	Attenuated macrophages by PCP
Anxa1	1.5247	5.37E-11	Anti-inflammatory in arthritis
Rgs2	1.8427	1.38E-09	Anti-inflammatory
Col7a1	1.8549	3.06E-08	Anti angiogenic
Ctss	1.5120	1.59E-07	JNK1/2 pathway modulator:<br>pro-osteoblast, anti-bone remodeling
Rras	1.5516	1.14E-05	Activates PI3K-Akt; Anti-angiogenic
Fcna	1.5001	1.34E-05	RA-associated locus polymorphism:<br>Negative correlation with SLE disease activity
Lamb2	1.7026	1.62E-05	Pro-angiogenic
Spn	1.5961	2.17E-05	Increases IL4R and IL4;<br>Anti-monocyte and anti-T cell; adhesion; Inhibits NF-kB activation;<br>T cell-constraining activity
Pdzd2	1.8577	6.25E-05	Response to ant-TNF treatment locus
Glipr2	1.7753	1.51E-04	Modulates cytokine production; Negative regulator of autophagy
Tnfrsf17	1.7698	3.57E-04	Immunosuppressing

**Downregulated**			
	**Fold Change**	**Padj**	**RA-relevant roles/functions**
Arid3a	1.6710	3.30E-12	Marker of CD19+ B lymphocytes
Cd80	1.6373	1.19E-05	Therapeutic target for RA
Cxcl10	1.7267	8.51E-05	Marker for RA disease and activity
Cxcl2	1.7056	1.55E-04	RA associated;<br>NF-kB-mediated osteoclastogenesis
Cxcr3	1.5758	1.84E-03	CXCL10 receptor, marker for RA disease and activity
Fam102a	1.8050	2.58E-12	Pro-osteoclastogenic
Fcrl1	1.5430	3.07E-11	Association with RA
Ifi44l	2.2361	1.04E-10	Interferon-inducible gene associated with RA
Il21r	1.7233	1.41E-04	RA pathogenesis
Myo1d	1.7571	3.22E-09	Pro-osteoclastogenic
Nfkbiz	1.5678	1.80E-04	Th17-differentiating transcription factor
Osgin1	1.7734	1.72E-08	Oxidative stress inducible autophagy
Prkca	1.6536	6.72E-10	Pro-angiogenic
Rsad2	1.7053	1.51E-04	RA-associated and type I interferon response gene
Slc40a1	2.1273	1.28E-09	Pro-inflammatory
Tnf	1.7878	3.91E-07	Therapeutic target for RA;<br>Activation of osteoclasts
Traf3ip3	1.6060	1.75E-09	Regulator of T and B cell development

Supplemental Table S2: Notable upstream regulators with RA relevance

S2A. Upstream regulators for 65-79*SE under M1 polarizing conditions

Upstream Regulator	RA-relevant functions	Predicted	z-score	p-value
STAT3	Pro-Th cells in RA; Pro-arthritis in mice; Pro-synoviocyte survival in RA	**Activated**	2.03	3.51E-35
JUN	Overexpressed in RA; Pro-artritis in mice	**Activated**	2.874	1.45E-21
EGR1	Pro-osteoclastogenic; NF-κB activator	**Activated**	3.129	4.29E-19
SP1	IL-1a activator; Induction of CCL2 expression	**Activated**	2.156	3.14E-17
EP300	Induction of CCL2 and IL-6 expression: Mediates IL-1β pro-inflammatory effect on RA synoviocytes	**Activated**	2.05	3.61E-17
RELA	Pathogenic factor in RA; Pro-arthritis in mice; Pro-osteoclastogenic	**Activated**	4.198	3.06E-15
CEBPB	Pro-inflammatory	**Activated**	3.455	3.27E-15
NUPR1	Pro-IL-1β-mediated expression of MMP13; Activates the NF-κB pathway	**Activated**	2.494	5.08E-15
CTNNB1	Activates RA synoviocytes; Pro-osteoclastogenic	**Activated**	3.661	7.14E-12
SMAD3	Pro-osteoclastogenic	**Activated**	3.119	1.71E-11
REL	Pathogenic factor in RA; RA susceptibility locus	**Activated**	3.323	7.71E-11
CEBPA	Inhibits M2 macrophage differentiation	**Activated**	2.812	9.03E-11
HIF1A	Pro-inflammatory in RA; Pro-angiogenic in RA; Activates PAD2-mediated protein citrullination in RA	**Activated**	2.42	9.62E-11
KLF2	Pro-M2 macrophage differentiation; Anti-arthritis in mice; Anti-angiogenic	**Inhibited**	-2.166	1.77E-10
FOXP3	Pro-Treg; RA-protective	**Inhibited**	-2.96	1.78E-09
XBP1	Anti-inflammatory; NF-κB inhibitor	**Inhibited**	-2.048	5.7E-10

S2B. Upstream regulators for 65-79*PE under M1 polarizing conditions

Upstream Regulator	RA-relevant functions	Predicted	z-score	p-value
SREBF1	Pro-inflammatory; Over-expressed in RA synoviocytes; Pro-angiogenic	**Inhibited**	-2.034	2.61E-10
KEAP1	Pro-inflammatory in basal conditions by promoting degradation of Nrf2	**Inhibited**	-2.191	5.1E-07
MITF	Pro-osteoclastogenic	**Inhibited**	-2.814	0.000165
SOX11	Pro-angiogenic	**Inhibited**	-2.292	0.000202
E2F1	Pro-osteoclastogenic; Pro-angiogenic	**Inhibited**	-2.353	0.000401
NFKBIZ	Pro-Treg; Anti-inflammatory: Enhances IL10 expression	**Activated**	2.122	0.00178
MTA1	Modulates cytokine networks in RA; Anti-inflammatory	**Activated**	2.378	0.00938

TARDBP	Inhibits NF-κB activity; Anti-inflammatory	**Activated**	2.236	0.0301
XBP1	Anti-inflammatory; NF-κB inhibitor	**Activated**	2.401	0.0492

S2C. Upstream regulators for 65-79*SE under M2 polarizing conditions

**Upstream Regulator**	**RA-relevant functions**	**Predicted**	**z-score**	**p-value**
EGR1	Over-expressed in RA synoviocytes; Pro-inflammatory in RA synoviocytes; Pro-angiogenic	**Activated**	2.229	2.36E-06
EPAS1	Pro-angiogenic	**Activated**	2.382	0.00396
GLI1	Pro-inflammatory cytokines in RA; Pro-RA synoviocye proliferation	**Activated**	2.371	0.0148
IRF3	Activates RA synoviocytes; Activates antibody production by B cells; Pro-M1; Pro-angiogenic	**Activated**	2.414	0.000152
IRF7	Pro-M1; Pro-arthritogenic in CIA mice	**Activated**	2.236	0.0257
TCF3	Inhibits the pro-RA β-catenin/Wnt pathway	**Inhibited**	-2	0.00555
RUNX1	Anti-osteoclast; Anti-angiogenesis	**Inhibited**	-2	0.00947

S2D. Upstream regulators for 65-79*PE under M2 polarizing conditions

**Upstream Regulator**	**RA-relevant functions**	**Predicted**	**z-score**	**p-value**
STAT6	Pro-M2; Anti-arthritis in mice	**Activated**	2.075	1.84E-11
KLF2	Pro-M2; Anti-arthritis in mice; Anti-angiogenic	**Activated**	2.401	0.0000233
NFATC2	Pro-osteoclastogenic; Pro-angiogenic	**Inhibited**	-2.091	1.82E-11
IRF5	Pro-inflammatory; RA-associated; RA risk locus; Associated with erosive RA; Pro-arthritis in mice	**Inhibited**	-2.362	0.0000356
EZH2	Over-expressed in RA synovium; Pro-osteoclastogenic; Pro-angiogenic	**Inhibited**	-2.601	0.000131
GATA6	Pro-angiogenic	**Inhibited**	-2.013	0.000144
KLF6	Pro-M1 and pro-inflammatory; Pro-angiogenic	**Inhibited**	-2.131	0.00184
IRF3	Activates RA synoviocytes; Activates antibody production by B cells; Pro-M1; Pro-angiogenic	**Inhibited**	-2.624	1.84E-07
IRF7	Pro-M1; Pro-arthritogenic in CIA mice	**Inhibited**	-2.115	2.74E-09

Purple font denotes pro-inflammatory or pro-RA regulators; green font denotes anti-inflammatory or anti-RA regulators.

Supplemental Table S3: List of reagents used in this study.

Reagent or Resource	Source	Category numbers
**Antibodies**		
Anti-mouse Akt	Cell Signaling Technologies (Danvers, MA)	Cat# 9272
Anti-mouse Phospho-Akt	Cell Signaling Technologies (Danvers, MA)	Cat# 9271
Anti-mouse Stat6	Cell Signaling Technologies (Danvers, MA)	Cat# 9362
Anti-mouse Phospho-Stat6	Cell Signaling Technologies (Danvers, MA)	Cat#56554
Anti-mouse SHIP1	Cell Signaling Technologies (Danvers, MA)	Cat# 2728
Anti-mouse Phospho-SHIP1	Cell Signaling Technologies (Danvers, MA)	Cat# 3941
Anti-mouse PTEN	Cell Signaling Technologies (Danvers, MA)	Cat# 9559
Anti-mouse Phospho-PTEN	Cell Signaling Technologies (Danvers, MA)	Cat# 9549
Anti-mouse beta-actin	Invitrogen (Waltham, MA)	Cat# BA3R
Anti-rabbit IgG HRP-linked	Cell Signaling Technologies (Danvers, MA)	Cat# 7074
Anti-mouse IgG HRP-linked	GE healthcare Lifesciences (Chicago, IL)	Cat# NA931
Anti-HLA-DR	Biolegend	Cat#307606
**Biological Samples**		
Fetal Bovine Serum	Corning (Tewksbury, MA)	Cat# 35-015-CV
Antibiotics (Pen Strep)	Gibco (Waltham, MA)	Cat# 15140-122
**Media**		
DMEM	Gibco (Waltham, MA)	Cat# 11965-092
DMEM	Gibco (Waltham, MA)	Cat# 11885-084
DMEM	Sigma (St. Louis, MO)	Cat# D5921
MEM Alpha	Gibco (Waltham, MA)	Cat# 12561-056
RPMI 1640	Gibco (Waltham, MA)	Cat# 11875-093
**Cells**		
RAW264.7	ATCC (Manassas, VA)	Cat# TIB-71
THP-1 cells	ATCC (Manassas, VA)	Cat# TIB-202
**Mice**		
HLA-DRB1*0401	Chela David	(*43*)
HLA-DRB1*0402	Chela David	(*44*)
**Chemicals, Reagents, peptides, and Recombinant Proteins**		
Recombinant Murine Il-4	R&D (Minneapolis, MN)	Cat# 404-ML-010
Recombinant Murine Il-13	R&D (Minneapolis, MN)	Cat# 413-ML-010
Recombinant Murine IFNγ	Peprotech (Rocky Hill, NJ)	Cat# 315-05
Recombinant Murine MCSF	Peprotech (Rocky Hill, NJ)	Cat# 315-02
Recombinant Human IFNγ	Peprotech (Rocky Hill, NJ)	Cat# 300-02
LPS	Sigma (St. Louis, MO)	Cat# L2630
65-79*SE (65-79*0401)	Bioworld (Dublin, OH)	(*72*)
65-79*PE (65-79*0402)	Bioworld (Dublin, OH)	(*72*)
PBS (Phosphate buffered saline)	Gibco (Waltham, MA)	Cat# 10010-023
Bovine Serum Albumin	Sigma (St. Louis, MO)	Cat# A7906
Sodium Pyruvate	Gibco (Waltham, MA)	Cat# 11360-070
DAF-2 DA	Sigma-Millipore (St. Louis, MO)	Cat# 251505

Novex™ 4-20% Tris-Glycine gels	Invitrogen (Waltham, MA)	Cat# XP04200BOX
RIPA Buffer	Sigma (St. Louis, MO)	Cat# R0278-50ml
cOmplete mini EDTA-free	Roche (Indianapolis, IN)	Cat# 11836170001
phosSTOP	Roche (Indianapolis, IN)	Cat# 04906845001
Trizol	Thermo Fisher (Waltham, MA)	Cat# 155596018
SuperSignal™ West Pico Plus ECL substrate	Thermo Scientific (Waltham, MA)	Cat# 34577
Ly294002	Sigma (St. Louis, MO)	Cat# L9908
Wedelolactone	Sigma (St. Louis, MO)	Cat# W4016
PF-4708671	Selleckchem (Houston, TX)	Cat# S2163
Phorbol 12-myristate 13-acetate	Sigma (St. Louis, MO)	Cat# P8139
See Supplementary materials for the Primer sequences used for qPCR	Integrated DNA Technology (Coralville, IA)	
Ghost Dye™ Violet 510	Tonbo	Cat# 13-0870-T100
**Critical commercial kits and assays**		
Mouse TNF-alpha DuoSet ELISA	R&D (Minneapolis, MN)	Cat# Dy-410-05
Mouse Il-12p70 DuoSet ELISA	R&D (Minneapolis, MN)	Cat# Dy-419-05
Mouse Il-6 DuoSet ELISA	R&D (Minneapolis, MN)	Cat# Dy-406-05
Arginase Activity Colorimetric Assay Kit	Biovision Inc. (Milpas, CA)	Cat# K755
Direct-zol™ RNA MiniPrep	Zymo Research (Irvine, CA)	Cat# R2052
RNeasy Plus Mini kit	Qiagen (Germantown, MD)	Cat# 74134
TruSeq RNA Sample Prep Kit	Illumina (San Diego, CA)	Cat# RS-122-2001
Fast SYBR™ Green Master Mix	Thermo Fisher (Waltham, MA)	
High Capacity cDNA Reverse transcription Kit	Thermo Fisher (Waltham, MA)	
TURBO DNA-free™ Kit	Invitrogen (Waltham, MA)	Cat# AM1907
Quantibody® Mouse Cytokine Array 1	Biolegend (San Diego, CA)	Cat# QAM-CYT-1
DC protein assay Kit	BioRad (Hercules, CA)	Cat# 5000112
**Software and Algorithms**		
Prism V8.1.0	GraphPad Software, Inc.	https://www.graphpad.com/scientificsoftware/prism/
ImageJ		https://imagej.nih.gov/ij/
Rsubread 1.5.0p3 & 1.6.1		https://bioconductor.org/packages/release/bioc/html/Rsubread.html
DESeq 2-1.16.1		http://bioconductor.org/packages/release/bioc/html/DESeq2.html
DAVID 6.8	https://david.ncifcrf.gov/	(*73*)

Ingenuity Pathway Analysis	Qiagen (Germantown, MD)	https://www.qiagenbioinformatics.com/products/ingenuity-pathway-analysis
KEGG: Kyoto Encyclopedia of Genes and Genomes	https://www.kegg.jp/kegg/	*Nucleic Acids Res*. 28, 27-30 (2000).
**Deposited data**		
RNA-seq data	This paper	GEO accession number GSE159821
**Other**		
Omega Lum C imaging system	Aplegen (San Francisco, CA)	
Synergy H1 Hybrid Multi-Mode Reader	Biotek (Winooski, VT)	https://www.biotek.com/products/detection-hybrid-technology-multi-mode-microplate-readers/synergy-h1-hybrid-multi-mode-reader/

Supplemental Table S4: List of primer sequences used for qPCR analysis.

Gene	Forward	Reverse
Ccl2	GAGGAAGGCCAGCCCAGCAC	TGGATGCTCCAGCCGGCAAC
Il-12p40	TCTTCTGCTTGGTTGGCTTT	CTCTGCGGGCATTTAACATT
Cxcl10	GGATGGCTGTCCTAGCTCTG	TGAGCTAGGGAGGACAAGGA
Nos2	CACCTTGGAGTTCACCCAGT	ACCACTCGTACTTGGGATGC
Arg1	GACCTGGCCTTTGTTGATGT	CAGCTCTTCATTGGCTTTCC
Ym	TAGTACTGGCCCACCAGGAA	AGACCTCAGTGGCTCCTTCA
Il-10	TGCACTACCAAAGCCACAAG	TAAGAGCAGGCAGCATAGCA
Il-6	CTTCACAAGTCCGGAGAGGA	TCCACGATTTCCCAGAGAAC
Tnfa	CTGGGACAGTGACCTGGACT	CTCCCTTTGCAGAACTCAGG
Il-1b	CAGGCAGGCAGTATCACTCA	TGTCCTCATCCTGGAAGGTC
Il-12p35	CATCGATGAGCTGATGCAGT	GAAGCAGGATGCAGAGCTTC
Il-23p19	CCAGCGGGACATATGAATCT	AGTCCTTGTGGGTCACAACC
Mgl2	ATCGCTTAGCCAATGTGCTT	TGGCCTCCAATTCTTGAAAC
Ccl17	AGTGGAGTGTTCCAGGGATG	TGGCCTTCTTCACATGTTTG
Adgre1	TGGAACACTGATGTGGAGGA	AGTTTGCCATCCGGTTACAG
Hprt	GCCCCAAAATGGTTAAGGTT	TTGCGCTCATCTTAGGCTTT

Unprocessed Western blot images

HLA-DRB1 Allelic Epitopes that Associate with Autoimmune Disease Risk or Protection Activate Reciprocal Macrophage Polarization

Vincent van Drongelen[1], Bruna Miglioranza Scavuzzi[1], Sarah Veloso Nogueira[1], Frederick W. Miller[2] Amr H. Sawalha[1,3], Joseph Holoshitz[1]*

[1] Department of Internal Medicine, University of Michigan, Ann Arbor, MI, 48109, USA.

[2] Environmental Autoimmunity Group, National Institute of Environmental Health Sciences, Research Triangle Park, NC, 27709, USA.

[3] Current address: Departments of Pediatrics and Internal Medicine, University of Pittsburgh, Pittsburgh, PA 15224, USA.

* Corresponding author. Email: jholo@med.umich.edu

Figure 3

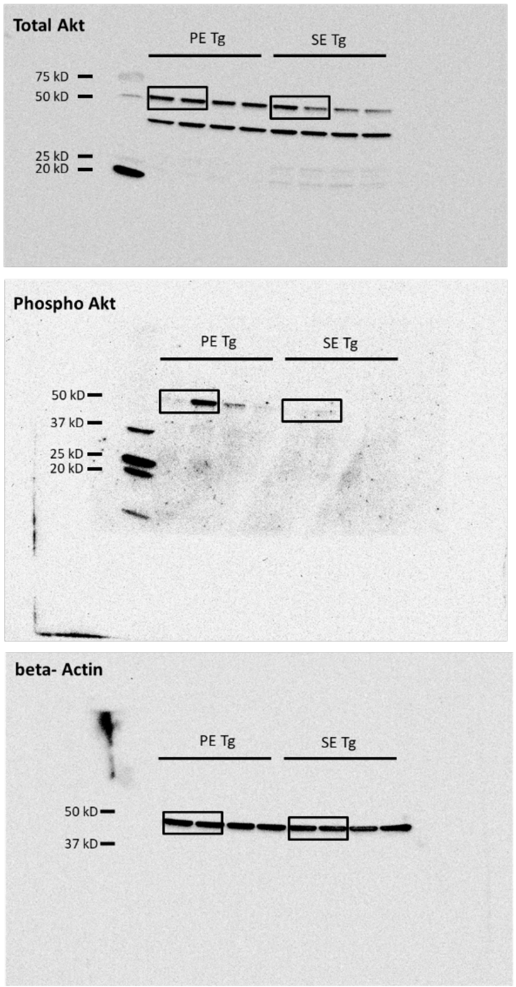

Figure 4

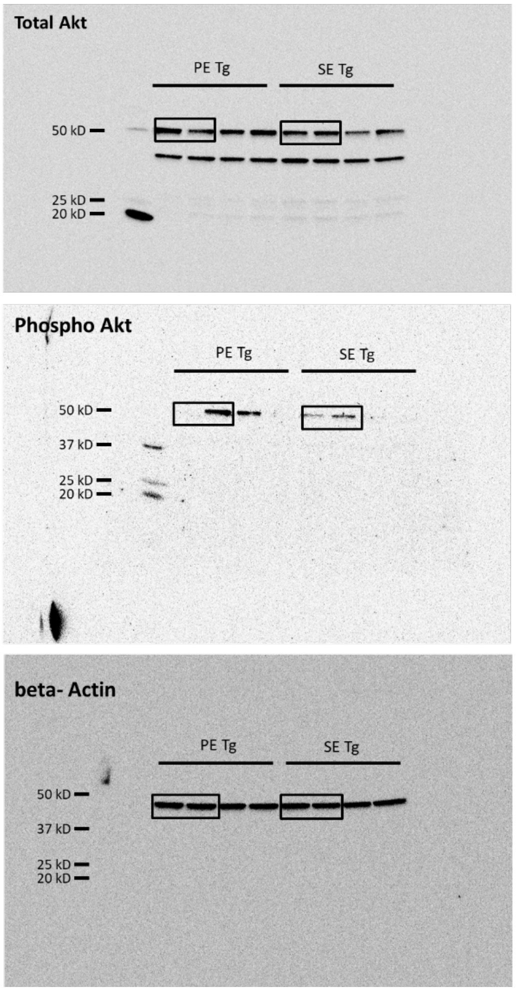

Figure 3 and Figure 4:

The membranes were:

1. probed with phosho-Akt antibody,
2. stripped, blocked and reprobed for total Akt antibody,
3. stripped, blocked and reprobed for beta-Actin antibody

Supplemental Figure 1

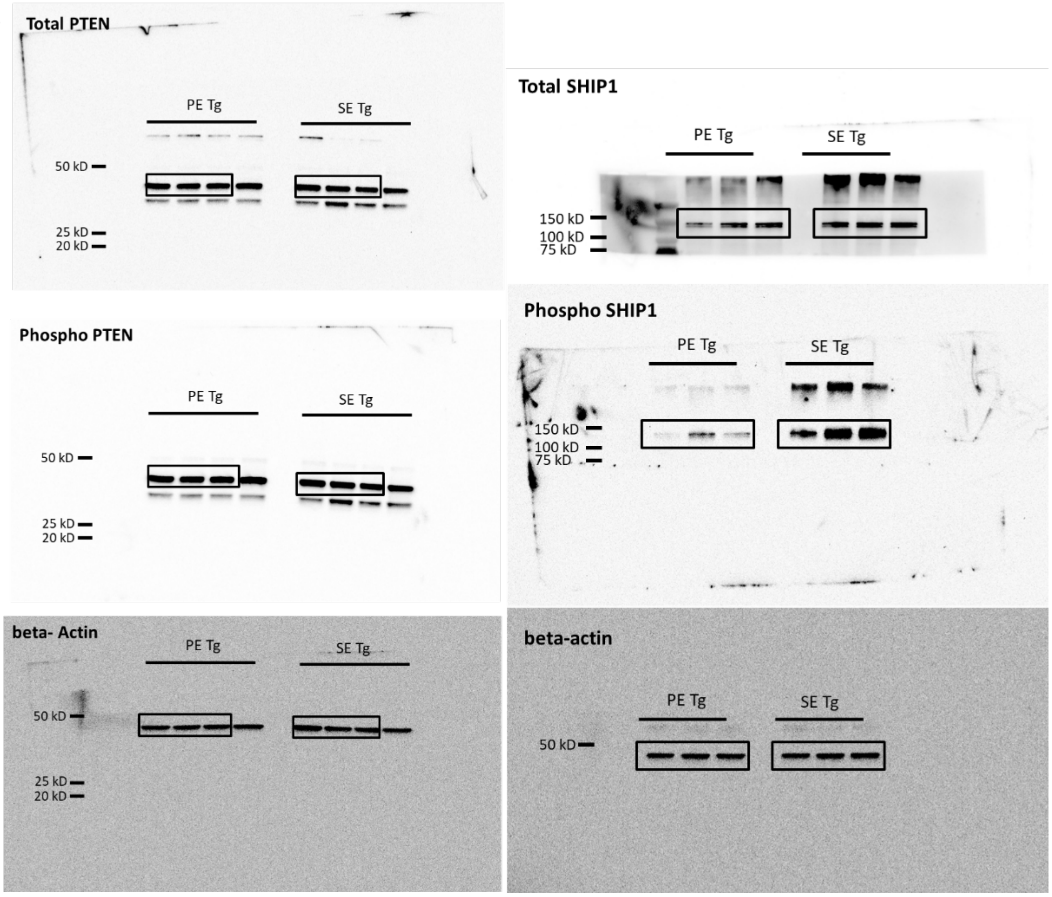

Supplemental Figure 1 PTEN
1. probed with phosho-PTEN antibody,
2. stripped, blocked and reprobed for total PTEN antibody,
3. stripped, blocked and reprobed for beta-Actin antibody

Supplemental Figure 1 SHIP
1. probed with phosho-SHIP antibody,
2. stripped, blocked and cut the higher part was reprobed for total SHIP antibody, the lower part was reprobed for beta-Actin antibody

Supplemental Figure 2

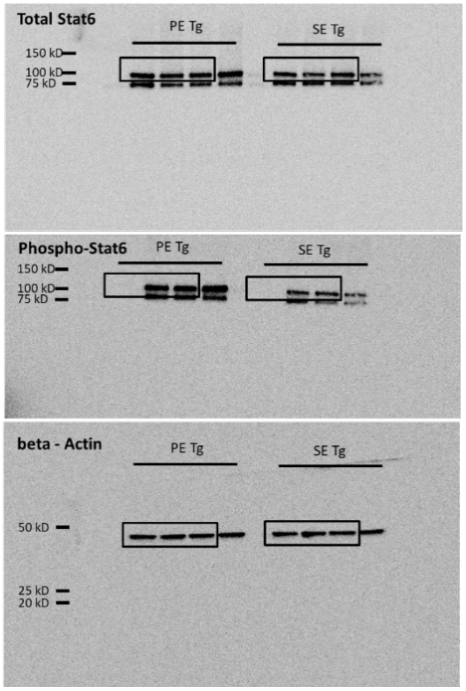

Supplemental Figure 2 Stat6
1. probed with phosho-Stat6 antibody,
2. stripped, blocked and reprobed for total Stat6 antibody,
3. stripped, blocked and reprobed for beta-Actin antibody.
